# Enhanced diagnostic interpretation of the MoCA using machine learning

**DOI:** 10.3389/fnins.2026.1679649

**Published:** 2026-02-20

**Authors:** Christian Gourdeau, Charles L. Gourdeau, Patrick J. Bernier, Robert Laforce

**Affiliations:** 1 Département de Physique, Cégep Limoilou, Québec City, QC, Canada; 2 Sciences, Informatique et Mathématique, Cégep Limoilou, Québec City, QC, Canada; 3 Services Gériatriques Spécialisés, CIUSSS de la Capitale-Nationale, Québec City, QC, Canada; 4 Clinique Interdisciplinaire de Mémoire du CHU de Québec, Québec City, QC, Canada

**Keywords:** Alzheimer, artificial intelligence, cognitive screening, machine learning, MoCA, dementia, cognitive charts, QuoCo

## Abstract

**Introduction:**

Artificial Intelligence (AI) is increasingly being integrated into clinical practice to optimize diagnosis in neurocognition. By capturing distinct cognitive signatures, this approach may offer a more precise alternative to the traditional interpretation of the Montreal Cognitive Assessment (MoCA) which often relies on a fixed cutoff score (26/30). We aimed to evaluate whether machine learning models, by integrating detailed MoCA subtest scores, demographic variables, and cognitive chart-derived metrics, can improve the detection of cognitive impairment and classification of dementia subtypes.

**Methods:**

We analyzed 38,746 clinical observations (17,188 unique individuals) from the National Alzheimer’s Coordinating Center database. Five supervised learning algorithms, Extreme Gradient Boosting (XGBoost), Random Forest, Support Vector Machine (SVM), Logistic Regression, and k-Nearest Neighbors (KNN), were trained using detailed MoCA subtest scores, demographic variables, and cognitive chart-derived metrics as predictors. To ensure generalizability of results and prevent data leakage, we applied a rigorous nested Repeated Grouped Cross-Validation strategy. Decision thresholds were optimized via the Youden Index on independent calibration sets, and model interpretability was ensured through SHAP value analysis.

**Results:**

Machine learning models consistently outperformed conventional approach. For the global detection of cognitive impairment, XGBoost achieved the best performance (Youden Index 0.61 vs. 0.54 for the standard cutoff). Regarding subtype classification, models demonstrated variable discriminative capacity depending on clinical homogeneity: primary progressive aphasia was best classified (Youden ≈ 0.77), followed by Lewy body dementia and Alzheimer’s disease, while vascular dementia remained more challenging to isolate. Feature importance analysis highlighted the Cognitive Quotient as a robust universal predictor, while pinpointing disease-specific drivers such as delayed recall for Alzheimer’s disease and verbal fluency for primary progressive aphasia.

**Conclusion:**

Our findings suggest interpretable machine learning enhances diagnostic utility of the MoCA, yielding superior accuracy compared to a fixed cutoff. By synthesizing individualized subtest profiles within a transparent framework, this approach offers a clinically actionable solution. It transforms the MoCA from a simple screening tool to a precision diagnostic aid, optimizing patient triage in the era of disease-modifying therapies.

## Introduction

The rapid expansion of artificial intelligence (AI) has led to increasing adoption across many areas of medicine where data driven approaches are being leveraged to support diagnostic interpretation, treatment personalization, and clinical decision making (Kantayeva et al., 2023; Topol, 2019). Within this broader landscape, machine learning is increasingly recognized as a central methodological approach for analyzing complex clinical datasets. In the field of neurocognitive disorders, machine learning based approaches have shown promise in addressing the substantial heterogeneity of clinical presentations by integrating cognitive, demographic, and clinical variables, thereby supporting earlier detection and improved diagnostic stratification (Mueller and Cammermeyer, 2023; Wang et al., 2024). Previous studies by James et al. (2021), Battista et al. (2017), and Zhan et al. (2015) have demonstrated that machine learning models applied to cognitive data can detect subtle patterns associated with cognitive impairment and outperform conventional clinical tools in classification and risk stratification tasks.

The aging population worldwide is driving a marked increase in the prevalence of dementia, making these conditions a major public health priority (Prince et al., 2015). Beyond their economic and societal burden, the recent emergence of disease modifying therapies, particularly monoclonal antibodies against amyloid accumulation such as donanemab and lecanemab, underscores the importance of timely and accurate diagnoses to optimize clinical benefit (Cummings et al., 2024; Sims et al., 2023; van Dyck et al., 2023).

**FIGURE 1:**
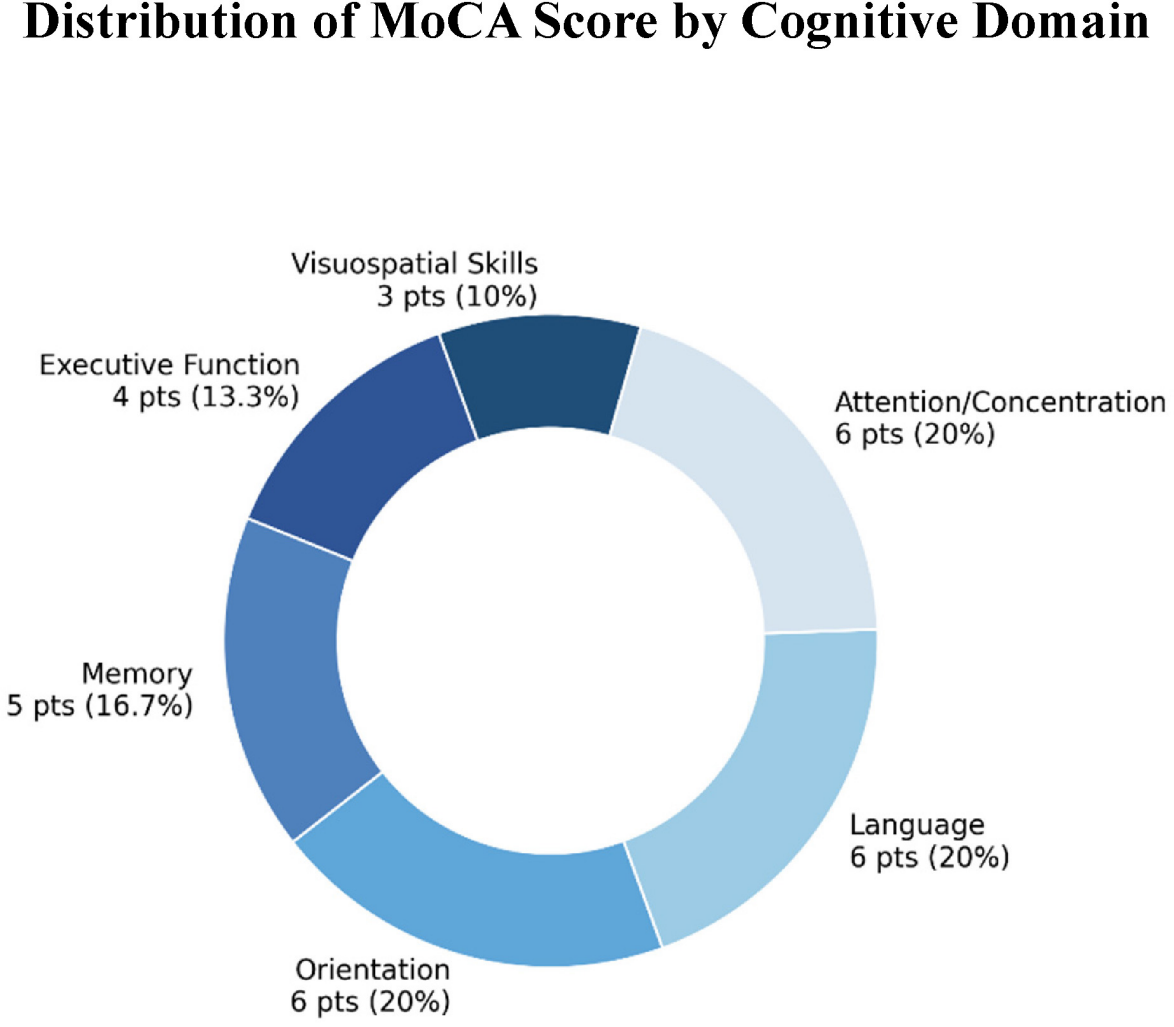
Scoring breakdown of the Montreal Cognitive Assessment (MoCA), indicating the maximum points and corresponding percentage of the total score assigned to each cognitive domain. This distribution reflects the relative weight of each domain in the global cognitive evaluation.

In clinical practice, the Montreal Cognitive Assessment (MoCA), developed by Nasreddine et al. (2005), remains a widely used screening tool for the early detection of mild cognitive impairment and for the characterization of cognitive deficits associated with neurocognitive disorders. It evaluates six major domains: memory, attention, executive functions, language, orientation, and visuospatial skills. The total score (out of 30 points) is derived from a weighted combination of subtests, each reflecting a specific cognitive domain, as illustrated in Figure 1.

Each cognitive domain contributes differently to overall performance and may carry varying clinical relevance depending on the underlying neurocognitive disorder. For instance, Cecato et al. (2016) reported that early impairments in memory recall, particularly in uncued recall tasks, are commonly observed in Alzheimer’s disease (AD). Skrobot et al. (2018) showed that vascular dementia (VD) is frequently associated with deficits in attention and executive functions, reflected in tasks such as serial subtraction and abstraction. In contrast, Coleman et al. (2016) described prominent impairments in verbal fluency, naming, and abstraction in behavioral variant frontotemporal dementia (bvFTD), with relative preservation of memory in early disease stages. Similarly, Biundo et al. (2016) emphasized that dementia with Lewy bodies (DLB), often characterized by visuospatial difficulties and cognitive fluctuations, is associated with poorer performance on clock drawing tasks and variability linked to demographic factors such as gender. Finally, Gorno-Tempini et al. (2011) highlighted the language-predominant cognitive profile of primary progressive aphasia (PPA), distinguishing it through specific deficits in repetition and verbal fluency. Taken together, these findings highlight the importance of analyzing MoCA sub scores rather than relying exclusively on the total score.

Interpreting MoCA scores using a fixed cutoff, most commonly 26/30, fails to adequately account for the well-established influence of demographic factors such as age and education. Carson et al. (2018) and Malek-Ahmadi et al. (2015) demonstrated that younger and more highly educated individuals may achieve scores within the normal range despite early cognitive impairment, whereas older adults or individuals with lower educational attainment may be misclassified as cognitively impaired despite normal aging. To refine MoCA-based assessments, Cognitive Charts were developed using large longitudinal datasets to contextualize cognitive performance relative to age and education level (Bernier et al., 2023; Gourdeau et al., 2025; Norton et al., 2024). Bernier et al. (2023) showed that incorporating two key indicators, the Cognitive Quotient (QuoCo), which represents an age-adjusted position of cognitive performance, and standardized age, which accounts for years of education, provides a more nuanced framework for interpreting cognitive test results. In the present study, these metrics are used as cross-sectional features to enhance the interpretation of individual cognitive profiles, rather than to model longitudinal change.

Many machine learning approaches have been applied to cognitive screening data to support diagnostic stratification and dementia subtype classification (James et al., 2021; Mueller and Cammermeyer, 2023; Wang et al., 2024). Building on this literature, supervised models such as Random Forest, Support Vector Machines, k-Nearest Neighbors, and gradient boosting methods including XGBoost can handle a large quantity of input features. In the present study, these features include MoCA subtest scores, demographic information, and cognitive chart-derived metrics, thereby extending prior work by integrating item-level cognitive information within a unified machine learning framework.

Altogether, this study had three main objectives. First, we aimed to distinguish cognitively normal individuals from those with cognitive impairment by comparing the performance of machine learning models integrating MoCA subtest scores, demographic variables (age and education), and cognitive chart-derived metrics (QuoCo and standardized age) with the conventional fixed MoCA cutoff approach. Second, we tested the ability of machine learning models to identify specific dementia pathologies including AD, VD, DLB, bvFTD, and PPA with respect to the overall study population, using a pathology versus rest classification framework. Finally, we identified the most influential cognitive and demographic variables associated with the detection of cognitive impairment and the differentiation of dementia subtypes, through the analysis of feature importance measures derived from the machine learning models.

## Materials and methods

### Data source and study sample

Data were obtained from the National Alzheimer’s Coordinating Center (NACC) database (versions 3.0 and 3.2), comprising 46,746 clinical visit records (Beekly et al., 2007). Among these visits, 5,657 did not include a Montreal Cognitive Assessment (MoCA) score and were excluded, leaving 41,089 visits with available MoCA data. To achieve the objectives of the present study, complete cognitive and demographic information was required for each clinical visit. Consequently, only visits with a fully administered MoCA and complete demographic data were retained for analysis. An additional 2,343 visits were excluded due to incomplete MoCA administration, most often attributable to clinical or functional limitations. This exclusion ensured that all analyses were conducted on a standardized and comparable set of MoCA subtests. The final analytic sample comprised 38,746 observations from 17,188 unique individuals.

Among the included observations, 21,534 were classified as cognitively normal and 17,212 as cognitively impaired, based on clinical diagnoses recorded in the NACC database. Demographic characteristics and cognitive performance measures across diagnostic groups are summarized in Table 1.

**TABLE 1:** Demographic and cognitive data by diagnosis.

Diagnosis	*N*	Female/male	Age	Education	MoCA
Normals	21534	64%/36%	72.67 ± 10.09	16.28 ± 2.75	26.41 ± 2.77
Cognitively impaired (all Dx)	17212	49%/51%	74.22 ± 9.89	15.76 ± 3.11	19.29 ± 6.44
Alzheimer	11463	51%/49%	75.66 ± 9.45	15.75 ± 3.12	18.18 ± 6.53
Vascular dementia	2867	52%/48%	78.20 ± 8.43	15.82 ± 3.36	20.69 ± 5.94
Lewy body dementia	560	18%/82%	73.20 ± 8.00	16.34 ± 3.03	15.50 ± 5.92
Behavioral variant frontotemporal dementia (bvFTD)	634	36%/64%	64.11 ± 9.16	15.73 ± 2.92	17.24 ± 6.98
Primary progressive aphasia (PPA)	569	46%/54%	67.08 ± 7.73	16.06 ± 2.51	13.64 ± 7.15

This table presents demographic characteristics and cognitive performance across diagnostic groups. Variables include the number of participants (N), percentage of females and males, age, years of education, and Montreal Cognitive Assessment (MoCA) scores, all reported as mean ± standard deviation (SD), if applicable. The Normal group comprises cognitively unimpaired individuals. The cognitively impaired (all Dx) group includes all participants diagnosed with at least one cognitive disorder, such as Alzheimer’s disease, vascular dementia, Lewy body dementia, bvFTD, or PPA, as well as other less frequent or unspecified neurocognitive conditions that were not analyzed separately. The total N for the cognitively impaired group does not equal the sum of the individual diagnostic categories. Some participants present with multiple co-occurring diagnoses and are therefore counted in more than one diagnostic subgroup, but only once in the overall cognitively impaired group.

### Study design and classification framework

Each clinical visit was treated as an independent observation, allowing individuals with multiple assessments to contribute more than one data point to the dataset. Although the NACC database includes longitudinal follow-up data, longitudinal cognitive trajectories were not analyzed. Importantly, all machine learning models were trained and evaluated using a patient-level data partitioning strategy to prevent information leakage, as described in detail in the training pipeline.

Diagnostic classification was first performed to distinguish cognitively normal individuals from cognitively impaired participants using a binary classification framework. The same pathology-versus-rest strategy was then applied to each dementia subtype, whereby individuals diagnosed with a specific condition (e.g., Alzheimer’s disease, vascular dementia, Lewy body dementia, behavioral variant of frontotemporal dementia, primary progressive aphasia) were contrasted against all remaining observations in the dataset. This approach enabled the identification of disease-specific cognitive patterns relative to the broader study population. To address these classification tasks, we evaluated five supervised machine learning algorithms. The following sections briefly describe the characteristics and implementation of each algorithm.

### Machine learning algorithms

#### Extreme Gradient Boosting (XGBoost)

Extreme Gradient Boosting is a gradient boosting ensemble method that builds decision trees sequentially, with each new tree correcting the errors of the previous ones (Chen and Guestrin, 2016). The algorithm incorporates regularization mechanisms and optimized computation, making it particularly effective for structured tabular data. In this study, XGBoost was implemented using the official XGBoost Python library.

Feature importance and model interpretability were assessed using SHapley Additive exPlanations (SHAP) (Lundberg and Lee, 2017). SHAP values quantify the marginal contribution of each feature to the model’s output, enabling consistent and theoretically sound interpretation of complex, non-linear models. This approach was applied to the XGBoost classifier, which was selected due to its strong performance on structured clinical data, its capacity to capture non-linear interactions among cognitive variables, and its robustness to feature collinearity and class imbalance (Chen and Guestrin, 2016). For tree-based ensemble models such as XGBoost, SHAP values are computed efficiently using the TreeSHAP algorithm (Lundberg et al., 2020).

#### Random Forest (RF)

Random Forest is an ensemble learning method based on decision trees, in which multiple trees are trained on random subsamples of the data and their predictions are aggregated (Breiman, 2001). This approach is robust against noise. The implementation used in this study is provided by the scikit-learn library (Pedregosa et al., 2011).

#### k-Nearest Neighbors (KNN)

This non-parametric algorithm classifies new samples based on their proximity to the k-Nearest Neighbors in the feature space (Cover and Hart, 1967). Its performance is influenced by the choice of k and the distance metric. KNN is particularly effective when the data exhibit strong local structure. This algorithm was also implemented using the scikit-learn library (Pedregosa et al., 2011).

#### Support Vector Machine (SVM)

Support Vector Machine (SVM) aims to maximize the margin separating classes in the feature space (Cortes and Vapnik, 1995). In this study, a linear SVM formulation was employed via the LinearSVC implementation from scikit-learn (Pedregosa et al., 2011). Although this variant does not rely on an explicit kernel mapping, it remains a Support Vector Machine and is well-suited for high-dimensional datasets where class separation is not trivial. To obtain probability estimates required for threshold optimization since SVMs do not natively provide probabilistic outputs we applied Platt scaling (Platt, 1999), which fits a Logistic Regression model to the classifier’s output.

#### Logistic Regression (LR)

Logistic Regression is a statistical model for binary classification that estimates the probability of class membership through a sigmoid transformation of a linear combination of explanatory variables (Hosmer et al., 2013). Despite its conceptual simplicity, it provides a strong baseline for comparison with more complex machine learning models. The implementation used in this work is provided by scikit-learn (Pedregosa et al., 2011).

### Training pipeline

To rigorously train and evaluate the algorithms, we employed a Repeated Grouped Cross-Validation procedure. This approach is considered a gold standard for reliably estimating model performance, particularly when navigating the complexities of clinical datasets.

The methodology centers on partitioning the dataset into “folds,” which function as independent mini-samples consisting of distinct patient subsets of comparable size. This granular division allows for a rigorous rotation of data roles. During each iteration, specific folds are allocated to either the training set, the test set, or, where applicable, a calibration set. A strict mutual exclusivity is maintained: a single fold cannot belong to two different sets within the same iteration. Once an iteration is complete, the folds are cycled, with each mini-sample shifting to a new role for the subsequent round. This systematic rotation continues until every data point has completed the cycle, ensuring an exhaustive and unbiased analysis of the entire dataset.

To manage data heterogeneity, we implemented a stratification strategy to ensure that the specific proportions of the original dataset are strictly maintained within each mini-sample. By enforcing identical ratios such as the balance between diagnostic classes or key demographic distributions across every fold, we eliminate the risk of performance variance caused by unrepresentative subsets. This structural consistency ensures that each evaluation cycle is performed on a fold that mirrors the global characteristics of the total population.

The “grouped” nature of this strategy is fundamental to maintaining the statistical independence of observations. In clinical environments, multiple measurements often originate from a single patient. According to Saeb et al. (2017), failing to account for this hierarchical structure introduces a high risk of “data leakage.” In such cases, the model may inadvertently memorize individual-specific traits rather than learning generalizable pathological signatures. By ensuring that all data from a single patient are confined to a single fold, we guarantee that a subject used during the training phase never appears in the testing phase. This methodological rigor is supported by Little et al. (2017), who show that such strict separation is the only viable method for obtaining an unbiased estimate of a model’s ability to generalize to new patient populations.

**FIGURE 2:**
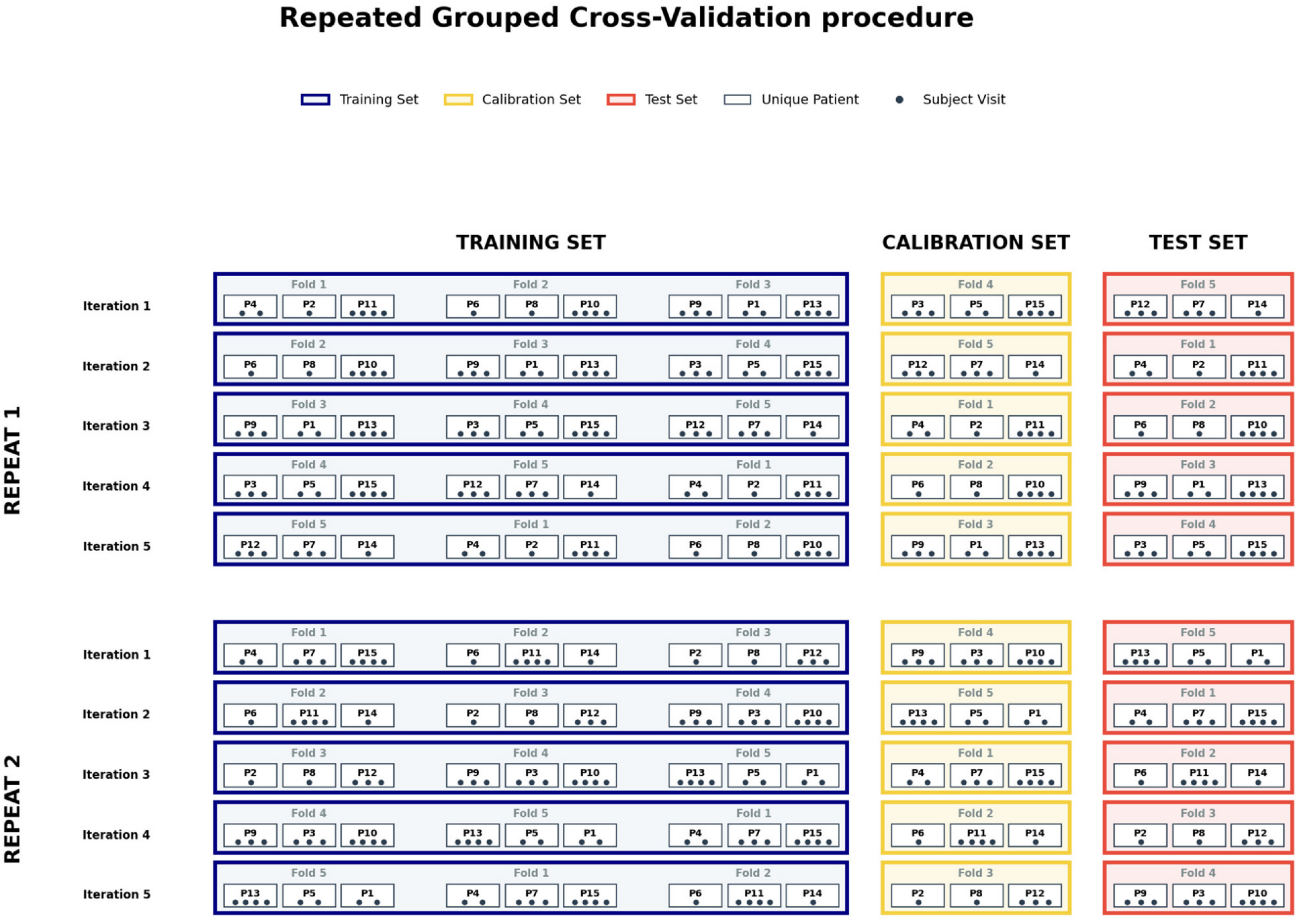
Schematic illustration of the Repeated Grouped Cross-Validation procedure. The diagram illustrates the data partitioning strategy ensuring strict independence between sets. Observations are grouped by unique patient (P1, P2, etc.) rather than by individual visit (black dots), ensuring that all data from a single subject remain confined within a single fold to prevent data leakage. This is for explanation purposes, in real analysis, it extends to 17,188 unique patients (P17188) and 6 repeats. At each iteration, the folds rotate between three functional sets: the training set (blue, 3 folds), the calibration set used for threshold optimization (yellow, 1 fold), and the independent test set (red, 1 fold). The procedure is repeated for a total of n repetitions with distinct random partitions to ensure robustness and reproducibility of performance estimates.

Finally, the procedure is “repeated,” meaning the entire partitioning process is executed multiple times with different random distributions. This repetition serves to smooth out variations caused by the randomness of the initial split. By combining these three dimensions k-fold partitioning, proportional grouping, and iterative repetition we achieve a highly representative evaluation of the model’s real-world performance, significantly minimizing the risks of overfitting and erroneous conclusions (Figure 2).

Building upon this framework, the dataset was subjected to a Repeated Grouped Cross-Validation scheme with five folds and six repetitions, resulting in a total of 30 independent outer iterations. One fold was reserved for testing, one fold for calibration, and the remaining three folds for model training, corresponding respectively to 20% test, 20% calibration, and 60% training data.

To address class imbalance, model-specific strategies were implemented to ensure robust generalization and mitigate majority-class bias. XGBoost utilized dynamic scale_pos_weight adjustments, an approach validated by Chen and Guestrin (2016) for effectively penalizing minority class misclassifications. Random Forest, Support Vector Machine, and Logistic Regression employed automatic class weighting (class_weight = “balanced”), a cost-sensitive learning method supported by King and Zeng (2001) as a superior alternative to resampling for maintaining data integrity in rare-event modeling. Conversely, the k-Nearest Neighbors algorithm utilized minority class oversampling prior to training, aligning with Chawla et al. (2002), who demonstrated that increasing local density is essential for defining clear decision boundaries in distance-based learners.

For each outer iteration, hyperparameters were optimized using either Optuna (Akiba et al., 2019) or a Grid Search approach, depending on the algorithm considered. To ensure a robust and unbiased evaluation of hyperparameter configurations, the training set of the current outer iteration was further subdivided using a nested Repeated Grouped Cross-Validation consisting of five folds and two repetitions, yielding 10 inner iterations per configuration. This nested architecture is essential to prevent “optimization bias,” as it ensures that hyperparameter tuning is performed strictly within the training data without any exposure to the calibration or test sets of the outer loop (Varma and Simon, 2006). Once the optimal hyperparameters were identified, the algorithm was retrained on the entire training set of the current outer iteration. The resulting model was then applied to the independent calibration set to determine the optimal decision threshold. Rather than relying on a default threshold (e.g., 0.5), we identified the optimal cutoff by maximizing the Youden Index.

This was calculated empirically by sorting the predicted probabilities and evaluating every possible split point to find the coordinate that maximizes the vertical distance between the Receiver Operating Characteristic (ROC) curve and the chance line, following the method described by Fluss et al. (2005). This approach allows the classifier’s operating point to be adjusted to better reflect clinical priorities, such as balancing false positives and false negatives. As noted by Hernández-Orallo et al. (2012), performing this threshold optimization on an independent calibration set is essential to ensure stable and clinically meaningful decision rules, particularly in imbalanced or high-stakes medical settings.

**FIGURE 3:**
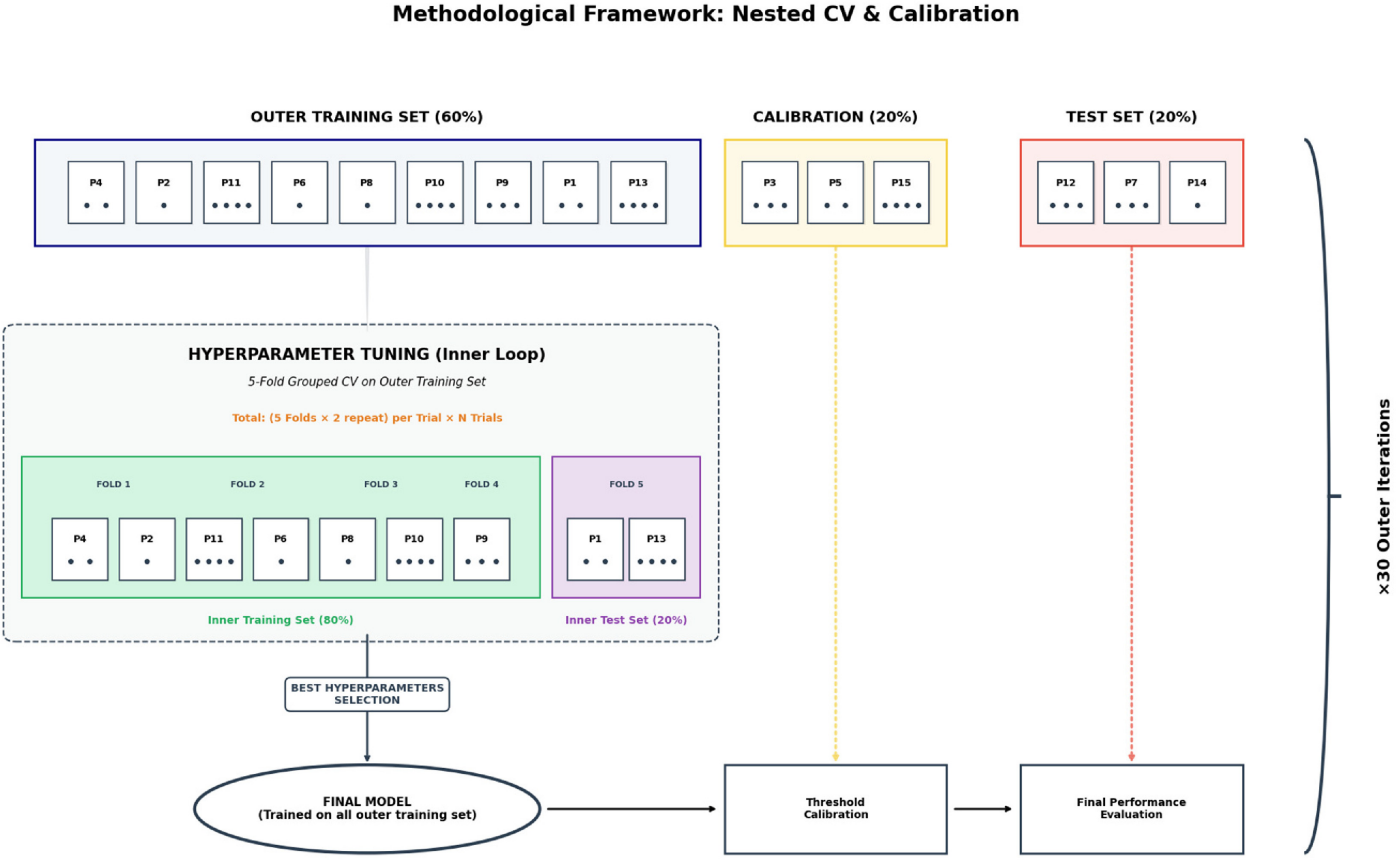
Methodological framework for nested cross-validation and model calibration. The diagram depicts the rigorous evaluation pipeline used for each of the 30 outer iterations. The dataset is partitioned into three distinct subsets: an outer training set (60%), a calibration set (20%) for threshold optimization, and a strictly held-out test set (20%) for final performance evaluation. Hyperparameter tuning is performed exclusively within the training set using an inner nested cross-validation loop to avoid optimization bias. Once optimal parameters are identified, the model is retrained on the full outer training set, calibrated to maximize the Youden Index on the independent calibration set, and finally assessed on the unseen test set to ensure an unbiased estimation of generalization performance.

After calibration, the fully trained model was finally evaluated on the held-out test set, which remained completely unseen throughout both hyperparameter optimization and threshold selection, thereby providing an unbiased estimate of real-world performance. The complete methodological framework, distinguishing between the inner optimization loop and the outer evaluation steps, is illustrated in Figure 3. This entire procedure was repeated 30 times for each algorithm and diagnostic category, corresponding to the 5-fold, 6-repeat outer cross-validation scheme. By aggregating results across these repetitions, we reduced the variance induced by random data partitioning, yielding a highly reliable estimate of model performance under realistic clinical deployment conditions, consistent with the recommendations of Krstajic et al. (2014).

## Results

### Overall algorithm performance

Across all classification tasks, ensemble-based tree models demonstrated strong and consistent performance. XGBoost emerged as the top performing algorithm in nearly all diagnostic categories, achieving the highest area under the ROC curve across all tasks and the highest or near highest Youden Index in most settings. The only exception was behavioral variant frontotemporal dementia, where XGBoost ranked second on the Youden Index, narrowly behind Logistic Regression. Differences among the three leading models XGBoost, Random Forest, and Support Vector Machines remained consistently small across diagnostic categories. Logistic Regression showed competitive performance in selected settings, while k-Nearest Neighbors consistently underperformed across all tasks. Receiver operating characteristic curves illustrating the diagnostic performance of the best-performing model (XGBoost) across classification tasks are provided in Supplementary Table 1.

### Global classification of cognitive impairment: comparison with the MoCA cutoff

For the global classification distinguishing cognitively normal individuals from those with cognitive impairment, supervised learning models consistently outperformed the conventional MoCA cutoff based approach. The standard cutoff score of 26/30 yielded a Youden Index of 0.539, whereas the best performing machine learning model achieved a higher Youden Index of 0.607 (Table 2). Among all evaluated algorithms, XGBoost provided the strongest overall performance, also achieving the highest AUC (0.882). This performance was characterized by a sensitivity of 0.744 and a specificity of 0.863, reflecting a strong ability to identify cognitively impaired individuals while maintaining accurate classification of cognitively normal participants. Predictive values were well balanced, supporting the use of this approach for first line cognitive screening.

**TABLE 2:** Classification performance by diagnosis.

*Algorithm*	Youden	Sensitivity	Specificity	PPV	NPV	AUC
**Normal vs. cognitive impairment**						
*XGBoost*	0.607	0.744	0.863	0.814	0.809	0.882
*Random* Forest	0.603	0.750	0.853	0.803	0.810	0.880
*SVM*	0.602	0.745	0.856	0.806	0.808	0.877
*Logistic*	0.601	0.747	0.854	0.804	0.809	0.877
*KNN*	0.565	0.734	0.831	0.778	0.797	0.858
*MoCA cutoff < 26*	0.539	0.847	0.693	0.687	0.850	0.865
**Alzheimer’s disease**						
*XGBoost*	0.593	0.765	0.828	0.653	0.893	0.876
Random Forest	0.587	0.764	0.822	0.646	0.892	0.873
*SVM*	0.582	0.765	0.817	0.639	0.892	0.866
*Logistic*	0.582	0.759	0.823	0.644	0.890	0.867
*KNN*	0.537	0.755	0.782	0.596	0.884	0.842
**Vascular dementia**						
*XGBoost*	0.305	0.617	0.688	0.138	0.957	0.710
Random Forest	0.303	0.632	0.671	0.135	0.958	0.709
*SVM*	0.282	0.642	0.639	0.126	0.957	0.696
*Logistic*	0.281	0.648	0.633	0.125	0.958	0.695
*KNN*	0.164	0.693	0.471	0.098	0.856	0.609
**Lewy body dementia**						
*XGBoost*	0.648	0.834	0.815	0.062	0.997	0.905
Random Forest	0.636	0.842	0.794	0.058	0.997	0.900
*SVM*	0.634	0.814	0.820	0.063	0.997	0.899
*Logistic*	0.632	0.819	0.813	0.062	0.997	0.897
*KNN*	0.274	0.804	0.470	0.044	0.530	0.758
**Behavioral variant frontotemporal dementia**						
*Logistic*	0.586	0.825	0.761	0.055	0.996	0.877
*XGBoost*	0.582	0.787	0.795	0.062	0.996	0.885
Random Forest	0.579	0.775	0.804	0.063	0.996	0.878
*SVM*	0.576	0.810	0.766	0.056	0.996	0.876
*KNN*	0.106	0.872	0.234	0.029	0.264	0.708
**Primary progressive aphasia**						
*XGBoost*	0.772	0.889	0.883	0.104	0.998	0.954
Random Forest	0.762	0.888	0.873	0.098	0.998	0.948
*SVM*	0.750	0.886	0.865	0.094	0.998	0.949
*Logistic*	0.745	0.880	0.865	0.096	0.998	0.948
*KNN*	0.452	0.803	0.649	0.094	0.697	0.837

Performance of five machine learning models (XGBoost, Support Vector Machine, Random Forest, Logistic Regression, and k-Nearest Neighbors) for the classification of cognitive status and dementia subtypes. Classification tasks include distinguishing cognitively normal individuals from those with cognitive impairment, and identifying Alzheimer’s disease, Vascular dementia, Lewy body dementia, behavioral variant of frontotemporal dementia, and primary progressive aphasia among all individuals. Metrics reported include sensitivity, specificity, positive predictive value (PPV), negative predictive value (NPV), Youden Index, and area under the ROC curve (AUC).

### Diagnostic specific performance of the best performing models

Diagnostic performance varied across categories but remained clinically meaningful, as reflected by the Youden Index. The highest discrimination was observed for primary progressive aphasia, with a maximum Youden Index of 0.772, followed by Lewy body dementia (0.648). Alzheimer’s disease (0.593) and behavioral variant frontotemporal dementia (0.586) showed intermediate performance whereas vascular dementia was the most challenging diagnosis, with Youden Index values not exceeding 0.305.

Beyond overall discrimination, the operating characteristics of the best performing models showed consistent patterns. Sensitivity and specificity were generally well balanced for diagnoses with clearer cognitive signatures. Negative predictive values were uniformly high, typically exceeding 0.95 and often approaching 0.99 for less prevalent diagnoses, supporting reliable exclusion of non-cases. In contrast, positive predictive values varied substantially across categories, largely reflecting disease prevalence. Overall discriminative performance was further supported by area under the ROC curve values, which were highest for primary progressive aphasia, Lewy body dementia, and Alzheimer’s disease, but lower for vascular dementia.

### Alzheimer’s disease

In Alzheimer’s disease, XGBoost showed the highest overall performance, with a Youden Index of 0.593 and an AUC of 0.876. Sensitivity (0.765) and specificity (0.828) were well balanced, and predictive values were higher than for rarer diagnoses.

### Vascular dementia

Vascular dementia was the most difficult diagnosis to classify. XGBoost achieved the highest Youden Index (0.305) and an AUC of 0.710. Sensitivity (0.617) and specificity (0.688) were modest, while the negative predictive value remained high (0.957).

### Lewy body dementia

For Lewy body dementia, XGBoost achieved the best performance, with a Youden Index of 0.648 and an AUC of 0.905. Sensitivity and specificity were high (0.834 and 0.815), and the negative predictive value remained very high (0.997).

### Behavioral variant frontotemporal dementia

For behavioral variant frontotemporal dementia, Logistic Regression achieved the highest Youden Index (0.586), slightly exceeding XGBoost, which retained the highest AUC (0.885). Sensitivity was high (0.825), specificity reached 0.761, and negative predictive values remained consistently high (0.996).

### Primary progressive aphasia

Primary progressive aphasia showed the strongest classification performance. XGBoost achieved a Youden Index of 0.772 and an AUC of 0.954, with high sensitivity (0.889) and specificity (0.883). The negative predictive value reached 0.998, while the positive predictive value remained low due to low prevalence.

Model convergence and training stability were further examined for the XGBoost classifier. Training and validation log loss curves as a function of the number of boosting rounds are provided in Supplementary Table 2.

### Cognitive signatures and key predictors of dementia subtypes

After evaluating classification performance, a feature importance analysis was performed to identify the variables contributing most strongly to each classification task. The results are summarized in Table 3 using two complementary perspectives. The first presents the most influential predictors among all variables included in the models, encompassing demographic factors, cognitive chart derived metrics, and MoCA based measures. The second focuses exclusively on MoCA subtest items, highlighting the relative contribution of individual cognitive domains independently of demographic and derived parameters. Detailed SHAP-based visualizations illustrating feature importance for each classification task are provided in Supplementary Table 3, while a complementary heatmap summarizing the relative influence of key variables across diagnostic categories is presented in Supplementary Table 4.

**TABLE 3:** Most influential variables and MoCA subtests identified by SHAP analysis.

Diagnostic category	Top 5 most significant variables (all features)	Top 5 MoCA subtests only
Cognitively normal vs. cognitive impairment	1. QuoCo 2. Total MoCA score 3. Delayed recall without cue 4. Orientation month 5. Orientation day	1. Delayed recall without cue 2. Orientation month 3. Orientation day 4. Orientation place 5. Orientation date
Alzheimer’s disease	1. Delayed recall without cue 2. Total MoCA score 3. Orientation date 4. QuoCo 5. Orientation month	1. Delayed recall without cue 2. Orientation date 3. Orientation month 4. Orientation place 5. Orientation day
Vascular dementia	1. Standardized age 2. Total MoCA score 3. Chronological age 4. QuoCo 5. Registration	1. Registration 2. Verbal fluency 3. Letter tapping A 4. Trail Making 5. Serial 7 subtraction
Lewy body dementia	1. QuoCo 2. Sex 3. Total MoCA score 4. Registration 5. Clock numbers	1. Registration 2. Clock numbers 3. Delayed recall without cue 4. Naming 5. Verbal fluency
Behavioral variant frontotemporal dementia	1. QuoCo 2. Chronological age 3. Standardized age 4. Verbal fluency 5. Sex	1. Verbal fluency 2. Orientation month 3. Abstraction 4. Repetition 5. Orientation place
Primary progressive aphasia	1. QuoCo 2. Repetition 3. Verbal fluency 4. Standardized age 5. Abstraction	1. Repetition 2. Verbal fluency 3. Abstraction 4. Registration 5. Delayed recall without cue

The most significant variables identified by the model for distinguishing cognitively normal individuals from those with cognitive impairment, as well as for classifying specific types of dementia. The variables are ranked in descending order of importance based on their contribution to classification accuracy. The middle column presents the five most important variables among all those used in the models. The right column lists the five most important variables drawn exclusively from the individual MoCA sub-items used in each classification task.

Across all diagnostic categories, cognitive chart derived metrics emerged as dominant contributors. The Cognitive Quotient (QuoCo) consistently ranked among the five most influential variables for all classification tasks and was the top ranked predictor for the global distinction between cognitively normal and cognitively impaired individuals, as well as for Lewy body dementia, behavioral variant frontotemporal dementia, and primary progressive aphasia. Standardized age also played a prominent role, ranking among the most influential predictors for several dementia subtypes, highlighting the importance of age and education adjusted cognitive positioning in these profiles. The total MoCA score remained a recurrent and informative predictor across multiple classification tasks, appearing among the top contributors for the global classification, Alzheimer’s disease, vascular dementia, and Lewy body dementia, while not ranking within the top five predictors for behavioral variant frontotemporal dementia and primary progressive aphasia.

Distinct cognitive signatures were observed across dementia subtypes. For the global classification of cognitively normal versus cognitively impaired individuals, the Cognitive Quotient emerged as the most influential predictor, followed by the total MoCA score and uncued delayed recall. When the analysis was restricted to MoCA subtests, orientation items including month, day, place, and date consistently ranked among the most influential features. This pattern highlights the strong discriminative contribution of orientation-based items within this classification framework.

In Alzheimer’s disease, the model was characterized by a predominantly mnemonic and temporal profile. Uncued delayed recall emerged as the most influential predictor, followed by the total MoCA score and temporal orientation items, particularly date orientation.

For vascular dementia, the classification model exhibited a relatively diffuse predictive pattern. Multifaceted deficits and age-related factors predominated, with standardized age emerging as the most influential predictor, followed by the total MoCA score, chronological age, and the Cognitive Quotient. This ordering suggests heterogeneous cognitive involvement with a strong contribution of overall cognitive burden and demographic context. At the subtest level, immediate memory registration and verbal fluency emerged as the most discriminative features, followed by attention demanding tasks such as letter tapping, consistent with predominant executive and attentional dysfunction.

In Lewy body dementia, the Cognitive Quotient was the dominant predictor, with sex emerging as a major contributor. At the subtest level, immediate registration and uncued recall were prominent, while the clock drawing task focused on number placement ranked among the most influential predictors. This pattern supports the relevance of visuospatial and executive dysfunction in this condition.

The behavioral variant frontotemporal dementia model was strongly influenced by age related variables, including both chronological age and standardized age. Cognitive predictors were dominated by verbal fluency and orientation to month, followed by abstraction tasks, reflecting the predominance of executive and frontal lobe related impairment.

Primary progressive aphasia exhibited a distinct and highly specific language driven signature. The Cognitive Quotient emerged as the most influential overall predictor, followed by language focused measures. Repetition and verbal fluency ranked immediately behind QuoCo, surpassing standardized age, while abstraction was followed by uncued delayed recall, completing the sequence of the most influential predictors.

Across all diagnostic categories, a small subset of items consistently ranked among the least influential predictors in the SHAP based analyses. Orientation to city systematically occupied the lowest or near lowest ranks across models, indicating minimal contribution to classification decisions regardless of diagnostic group. Similarly, visuospatial construction items related to clock drawing details, specifically clock drawing hands and clock drawing contour, consistently ranked in the lower range of importance. Cube copying also showed persistently low ranks across most diagnostic categories, frequently appearing among the least influential variables. Collectively, these items rarely entered the upper tiers of variable importance and remained consistently positioned at the bottom of the SHAP ranking distributions across all classification tasks.

## Discussion

This study highlights the potential of machine learning approaches to enhance the diagnostic interpretation of the Montreal Cognitive Assessment for detecting cognitive impairment and classifying dementia subtypes. By integrating detailed MoCA subtest scores, demographic variables such as age and education, and cognitive chart-derived metrics including the Cognitive Quotient and standardized age, the proposed models demonstrated improved performance compared with the conventional MoCA cutoff score of 26 out of 30. Among the evaluated approaches, XGBoost achieved the strongest overall performance in almost all diagnostic categories, providing a more favorable balance between sensitivity and specificity than the fixed threshold strategy. To our knowledge, this is the first study to examine all MoCA items jointly and relate them to diagnostic performance. These findings are consistent with prior research emphasizing the limitations of fixed cutoff-based approaches (Carson et al., 2018; Malek-Ahmadi et al., 2015) and support the growing role of machine learning methods in the assessment of neurocognitive disorders (Kantayeva et al., 2023; Topol, 2019).

### Superior performance of machine learning models

Most of the evaluated machine learning models, which integrated detailed MoCA subtest scores, demographic variables, and cognitive chart-derived metrics, outperformed the conventional MoCA cutoff score of 26 out of 30 (Youden Index: 0.539) in distinguishing cognitively normal individuals from those with cognitive impairment. Among these approaches, XGBoost achieved the strongest overall performance in almost all diagnostic categories, reaching a Youden Index of 0.607 for global classification. Random Forest, Support Vector Machines, and Logistic Regression showed closely comparable performance, with Youden Index values near 0.60, whereas k-Nearest Neighbors consistently yielded lower discriminative performance, approaching that of the conventional cutoff strategy.

These findings underscore the value of a multidimensional approach that integrates MoCA subtest data, demographic variables, and cognitive chart metrics rather than relying on a single global threshold. This observation is consistent with previous studies by Carson et al. (2018) and Malek-Ahmadi et al. (2015), as well as earlier work by Nasreddine et al. (2005), which highlighted the limitations of fixed MoCA cutoffs that fail to account for demographic influences such as age and education. Cognitive charts, as proposed in prior work (Bernier et al., 2023; Gourdeau et al., 2025; Norton et al., 2024), directly address these limitations by contextualizing cognitive performance relative to demographic factors. More broadly, these results are consistent with growing evidence supporting the role of machine learning in dementia diagnostics (Kantayeva et al., 2023; Topol, 2019). The present study extends findings by James et al. (2021) by applying interpretable machine learning models to detailed MoCA subtest data, highlighting the added value of domain-specific cognitive information for early detection.

### Differential classification and diagnostic complexity

Beyond global cognitive impairment detection, the machine learning models demonstrated meaningful capacity to differentiate between major dementia subtypes. Across diagnostic categories, XGBoost consistently emerged as the best performing or near-best performing algorithm, achieving the highest Youden Index in almost all subtypes. However, classification performance varied substantially depending on the clinical homogeneity of the condition.

Primary progressive aphasia (PPA) demonstrated the strongest classification performance of all subtypes, followed closely by Lewy body dementia and Alzheimer’s disease. This likely reflects the distinct and relatively homogeneous cognitive signatures of these conditions, predominantly linguistic for aphasia and mnemonic for Alzheimer’s disease, which are well-captured by the model features. This could be surprising from a clinician point of view because the detection of PPA is often a great challenge by comparison to Alzheimer’s disease for non-expert.

In contrast, vascular dementia remained the most challenging diagnosis to classify, with modest Youden Index and AUC values. This lower performance reflects the inherent clinical heterogeneity of vascular dementia, which can manifest as diverse cognitive profiles depending on lesion location and severity. Furthermore, as noted by Skrobot et al. (2018), the diagnosis of vascular dementia in clinical practice relies heavily on neuroimaging and stroke history, variables that were absent from this dataset. The reliance on cognitive test scores alone is therefore a limiting factor for this specific subtype.

While no prior study has documented the accuracy of clinicians using specific items of the MoCA in isolation, current literature suggests that multimodal approaches integrating several data points yield superior diagnostic performance (Qiu et al., 2022). However, these comprehensive approaches are usually more costly and not readily available in all clinical settings.

### Cognitive signatures and model interpretability

The feature importance analysis based on SHAP values validated the model’s decision-making process by revealing cognitive signatures consistent with established clinical knowledge. Across all models, cognitive chart-derived metrics emerged as dominant contributors, with the Cognitive Quotient (QuoCo) ranking among the most influential variables for all diagnostic categories. This reinforces the value of contextualizing performance relative to demographic norms to detect subtle deviations (Bernier et al., 2023; Gourdeau et al., 2025; Norton et al., 2024).

Distinct cognitive profiles emerged for each subtype:

1. **Alzheimer’s Disease:** The model was driven primarily by memory and temporal orientation features. Uncued delayed recall emerged as the most influential predictor, followed by orientation to date and month. This hierarchy accurately reflects the hallmark episodic memory impairment and temporal disorientation described in the literature (Cecato et al., 2016).
2. **Primary Progressive Aphasia:** This diagnosis was characterized by a highly specific language-driven signature. Repetition and verbal fluency ranked immediately behind the Cognitive Quotient, surpassing standardized age, while memory-related tasks played a secondary role. This clear hierarchy confirms the linguistic specificity of primary progressive aphasia (Gorno-Tempini et al., 2011) and aligns with the rationale behind screening instruments specifically developed to capture these linguistic deficits, such as the Dépistage Cognitif de Québec (DCQ) (Laforce et al., 2018).
3. **Lewy Body Dementia:** The Cognitive Quotient and sex were dominant predictors, while subtest analysis highlighted immediate memory registration, uncued recall, and visuospatial executive components (clock number placement). This aligns with the known profile of visuospatial impairment and fluctuations in attention (Biundo et al., 2016).
4. **Behavioral Variant Frontotemporal Dementia:** Executive and language-related measures predominated, with verbal fluency and orientation to month emerging as key discriminators. Interestingly, naming did not rank among the most influential subtests, suggesting that executive dysfunction (fluency) is a more reliable marker than confrontation naming for this variant.
5. **Vascular Dementia:** Unlike other subtypes driven by distinct focal deficits, the classification model for vascular dementia relied predominantly on broad demographic and global cognitive indicators. Standardized age and the total MoCA score emerged as the strongest predictors, suggesting that this condition is characterized by a generalized cognitive decline closely tied to aging rather than a specific isolated domain impairment. In the absence of neuroimaging, the model complemented this global profile with markers of executive and attentional dysfunction (verbal fluency, letter tapping) and immediate memory registration. This reliance on a heterogeneous combination of global and subtest-level variables aligns with the inherent clinical variability of vascular dementia, which typically manifests as dysexecutive symptoms and processing speed deficits superimposed on a background of global decline, rather than a single signature deficit (Skrobot et al., 2018).

These cognitive profiles are consistent with the criteria commonly used by experts such as neurologists and neuropsychologists.

A complementary SHAP-based analysis identified several MoCA items with low discriminative value. Orientation to city, contour integrity and hand placement in the clock drawing task, as well as cube copying, consistently ranked among the least influential predictors across diagnostic categories. This pattern does not indicate that these items lack clinical relevance, but rather suggests that, within the context of multivariate machine learning models integrating a broad set of cognitive and contextual variables, they contribute less to diagnostic discrimination than other MoCA components. These items tend to rely on relatively coarse or near binary scoring, which may limit their incremental informational value once more sensitive measures are considered.

Importantly, machine learning models operate exclusively on the numerical scores assigned to each MoCA item and do not have access to the qualitative or structural characteristics of the original responses. For example, in the clock drawing task, the model does not “see” the drawing itself but only receives discrete subscores corresponding to contour integrity, number placement, and hand positioning. In contrast, experienced clinicians may extract additional diagnostic information from qualitative features such as global shape distortion, spatial organization, perseverations, or asymmetries even when the numerical score is identical.

In this context, the reduced influence of contour integrity and hand placement should not be interpreted as a lack of importance of the clock drawing task as a whole. Other components of this task, particularly number placement, demonstrated substantially higher discriminative relevance for specific diagnoses. Together, these findings highlight the heterogeneous informational content of individual MoCA items and underscore the complementary roles of item-level machine learning analysis and expert clinical interpretation when cognitive screening tools are used for diagnostic support.

## Clinical implications and translation

The rising global prevalence of dementia highlights the urgent need for accurate diagnostic strategies, particularly in the context of emerging disease-modifying therapies (Cummings et al., 2024; Prince et al., 2015; Sims et al., 2023; van Dyck et al., 2023). Our findings suggest that enhanced machine learning-based interpretation of the MoCA may support earlier identification of candidates for these therapies, while reducing misclassification between Alzheimer’s disease and potentially non-responsive conditions like frontotemporal dementia. We do not suggest that MoCA analysis should be use solely for diagnosis of neurocognitive disorders. We still emphasize the importance of complete clinical assessment.

Critically, for such tools to be adopted in clinical practice, they must be transparent. In this study, concerns regarding the “black box” nature of AI were addressed through the use of SHAP-based analyses, consistent with the unified approach to model interpretation proposed by Lundberg and Lee (2017). Unlike opaque model outputs, SHAP values provide transparent, patient-level explanations of variable contributions, thereby mitigating the interpretability barrier commonly cited in medical AI, as noted by Tjoa and Guan (2021). This transparency allows clinicians to verify that the model’s “reasoning” aligns with medical knowledge, fostering trust and responsible deployment.

From an implementation perspective, the consistently strong performance of XGBoost combined with this interpretability supports its potential clinical relevance. However, realizing this potential will require overcoming recognized challenges in deploying ML tools, such as integration into existing clinical workflows and validation in diverse real-world settings, as highlighted by Kelly et al. (2019).

## Limitations

Several limitations should be considered when interpreting these findings. First, the dataset was derived from a specialized clinical cohort, which differs from the general population in terms of disease prevalence, referral patterns, and demographic characteristics. This referral bias may limit the direct generalizability of the results to community based or primary care settings. In addition, the exclusion of observations with incomplete MoCA, age, or education data, while necessary to ensure analytical consistency, may have introduced further selection bias.

Second, although multiple visits from the same individuals were included in the dataset, the present study adopted a cross sectional design. Consequently, longitudinal changes in cognitive performance and individual trajectories of decline were not modeled. While patient level data partitioning prevented information leakage between training and test sets, the absence of explicit temporal modeling limits the ability to assess progression or conversion risk. Longitudinal approaches have previously been shown to provide complementary insights into cognitive decline trajectories (Kantayeva et al., 2023).

Third, the analysis relied exclusively on cognitive and demographic variables. This constitutes a limitation, particularly for heterogeneous conditions such as vascular dementia. The absence of complementary clinical information, including neuroimaging markers, documented vascular events, or fluid biomarkers, restricts the capacity of the models to capture underlying disease biology. Previous studies have demonstrated that integrating multimodal data can improve diagnostic accuracy (Qiu et al., 2020; Zhan et al., 2015). However, an explicit aim of the present work was to evaluate the capacity of machine learning models to interpret item-level MoCA performance as a decision support framework, rather than as a standalone diagnostic tool.

Fourth, the current classification framework grouped individuals based on diagnostic pathology (e.g., Alzheimer’s disease) regardless of clinical severity. By pooling Mild Cognitive Impairment (MCI) and dementia cases under the same diagnostic label to perform a “pathology-versus-rest” classification, the specific cognitive signatures that might distinguish prodromal stages from established disease were not independently analyzed. While this approach maximizes sample size and detection power for the pathology itself, it may mask subtle stage-specific variations in feature importance and limits our ability to draw conclusions regarding the model’s performance specifically within the MCI population.

## Methodological strengths

A major strength of this study lies in the breadth and rigor of its modeling framework. Rather than relying on a single algorithm, we implemented a comprehensive validation strategy to ensure the robustness and reproducibility of the identified cognitive signatures. Five distinct machine learning algorithms XGBoost, Random Forest, Support Vector Machines, Logistic Regression, and k-Nearest Neighbors were evaluated across six diagnostic contexts, including global cognitive impairment and five dementia subtypes.

Each model underwent systematic hyperparameter optimization using Optuna or grid search procedures. Model performance was assessed using stratified five-fold cross validation, repeated multiple times to minimize variance related to data partitioning. This intensive computational strategy involved the training and independent evaluation of thousands of models, ensuring that the observed performances were not driven by favorable splits or chance effects.

Importantly, the consistency of results across algorithms supports the stability of the identified cognitive signatures. The strong and reproducible performance observed for specific conditions, such as primary progressive aphasia and Alzheimer’s disease, reflects robust signal extraction rather than methodological artifacts. In addition, the use of SHAP based explainability enabled transparent quantification of the relative contribution of each variable, strengthening the clinical interpretability of the findings.

## Future directions

Several avenues for future research emerge from this work. Beyond the present framework, advances in natural language processing and large language models provide additional opportunities to extend the analysis of linguistic components of the MoCA, including verbal fluency and sentence repetition. These approaches have been shown to capture subtle semantic, syntactic, and acoustic markers of cognitive decline that are not reflected in conventional scoring and could further enhance subtype-specific discrimination (Agbavor and Liang, 2022).

To further refine diagnostic stratification, future work should also aim to analyze clinical stages separately. Investigating stage-specific machine learning models, for instance by training algorithms exclusively on MCI cohorts versus dementia cohorts, could uncover distinct cognitive markers for early versus late-stage disease. Such granular analysis would help determine whether the most influential MoCA subtests shift as neurodegeneration progresses, thereby providing more targeted clinical insights for early screening.

In parallel, future investigations will leverage the longitudinal structure of the NACC dataset to model individual cognitive trajectories over time. Incorporating temporal dynamics and rates of decline into machine learning frameworks represents a critical next step for improving predictive accuracy, particularly for identifying individuals at high risk of progression from mild cognitive impairment to dementia (Kantayeva et al., 2023).

Ultimately, validating these findings across more diverse populations and clinical settings will be essential to ensure robustness, equity, and broader clinical applicability (Prince et al., 2015). Expanding the approach to multimodal datasets while preserving interpretability will further support translation into real-world clinical decision support systems.

## Conclusion

This study has shown that the richness of MoCA subtest data, when leveraged by interpretable machine learning, far exceeds the diagnostic utility of the traditional total score. By integrating demographic characteristics, the cognitive chart-derived metrics and granular item-level performance, our models achieved high accuracy while maintaining interpretability through biologically validated feature importance. In the era of emerging disease-modifying therapies, where timely and precise identification of pathology is paramount, this framework provides a scalable solution to reduce misclassification and optimize patient triage. Future investigations integrating longitudinal trajectories and stratified analyses by clinical severity will allow these models to better mimic clinical reasoning, further solidifying the role of such AI-driven decision support tools in routine practice.

## Data Availability

The datasets presented in this study can be found in online repositories. The names of the repository/repositories and accession number(s) can be found below: NACC web site: https://naccdata.org/.
