# Supplementary material for: Enhanced diagnostic interpretation of the MoCA using machine learning
Source: Front Neurosci. 2026 Feb 20;20:1679649. doi: 10.3389/fnins.2026.1679649 (PMC12963294; doi:10.3389/fnins.2026.1679649)
Supplement: Supplementary file 1 [file Data_Sheet_1.pdf]

Supplementary material 3

SHAP Summary Plots illustrating feature importance for global cognitive impairment detection and dementia subtype classification.

These plots illustrate the impact of each feature on the XGBoost model's decision-making process for each classification task. Features are ranked from top to bottom in descending order of importance, based on the mean absolute SHAP value.

Note: For the *NORMCOG* classification task, the positive class label was inverted for modeling purposes; therefore, a positive SHAP value corresponds to cognitive impairment rather than normal cognition.

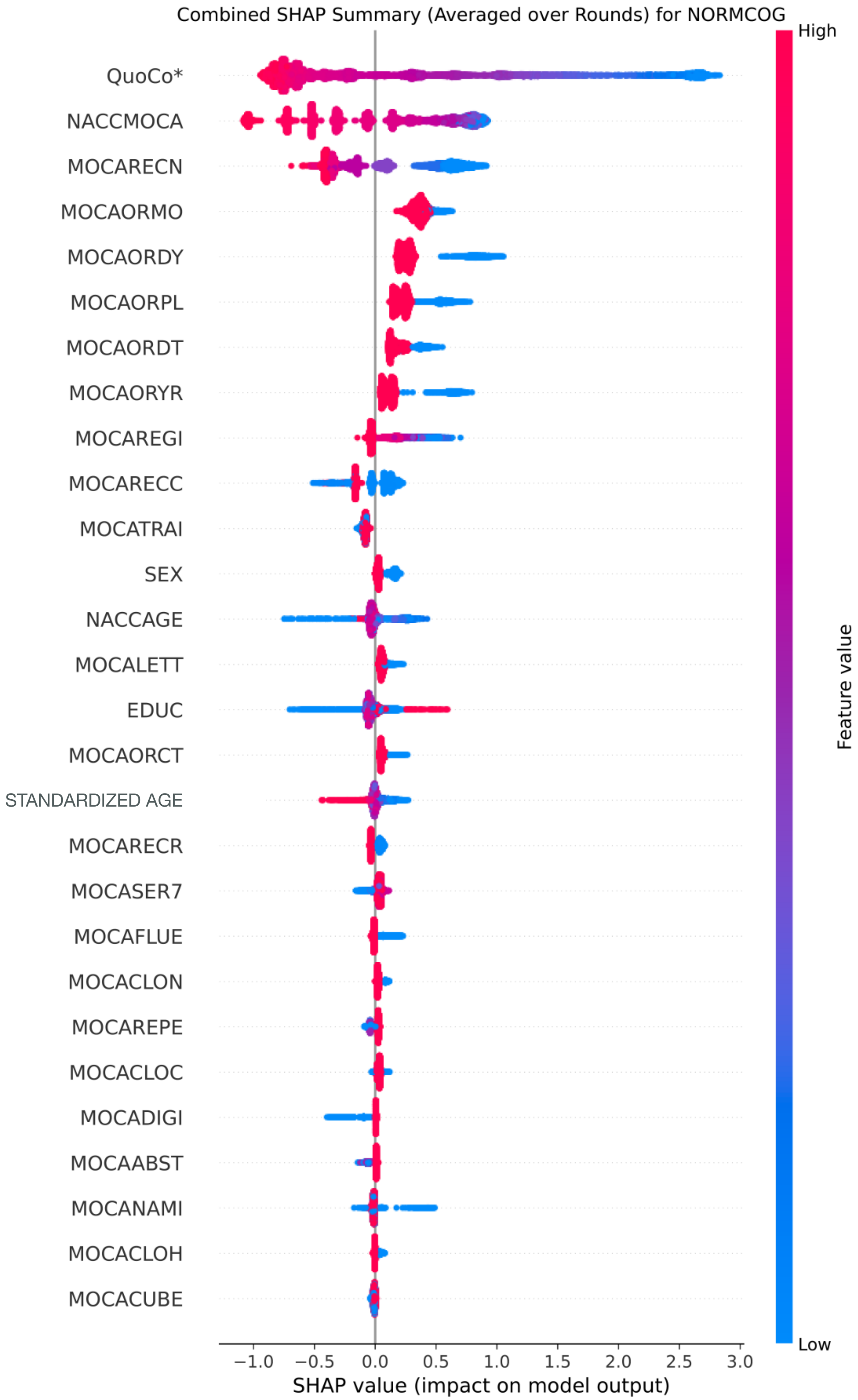
Combined SHAP Summary (Averaged over Rounds) for NORMCOG
QuoCo*
NACCMOCA
MOCARECN
MOCAORMO
MOCAORDY
MOCAORPL
MOCAORDT
MOCAORYR
MOCAREGI
MOCARECC
MOCATRAI
SEX
NACCAGE
MOCALETT
EDUC
MOCAORCT
STANDARDIZED AGE
MOCARECR
MOCASER7
MOCAFLUE
MOCACLON
MOCAREPE
MOCACLOC
MOCADIGI
MOCAABST
MOCANAMI
MOCACLOH
MOCACUBE
−1.0
−0.5
0.0
0.5
1.0
1.5
2.0
2.5
3.0
SHAP value (impact on model output)
High
Low
Feature value

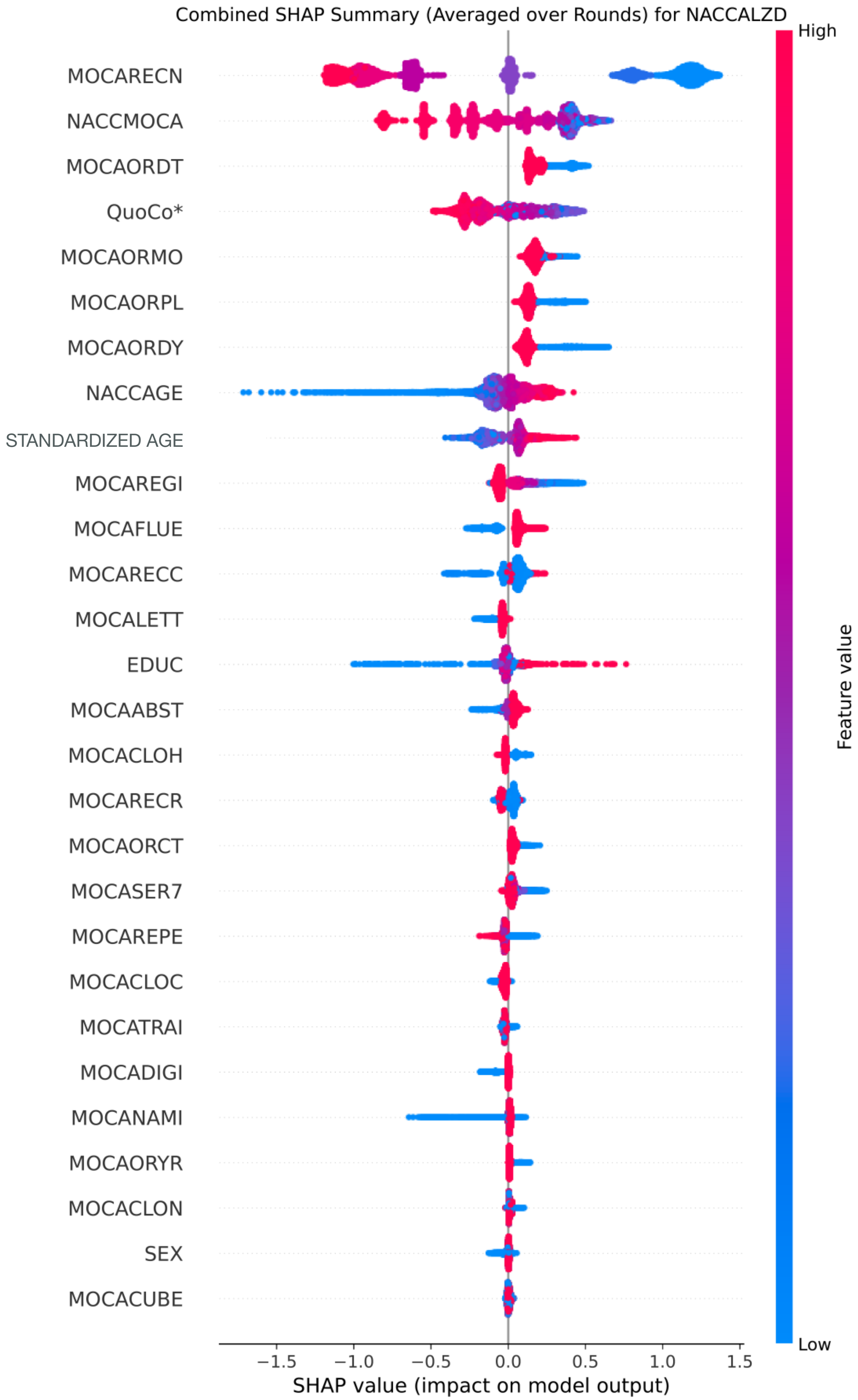
Combined SHAP Summary (Averaged over Rounds) for NACCALZD
MOCARECN
NACCMOCA
MOCAORDT
QuoCo*
MOCAORMO
MOCAORPL
MOCAORDY
NACCAGE
STANDARDIZED AGE
MOCAREGI
MOCAFLUE
MOCARECC
MOCALETT
EDUC
MOCAABST
MOCACLOH
MOCARECR
MOCAORCT
MOCASER7
MOCAREPE
MOCACLOC
MOCATRAI
MOCADIGI
MOCANAMI
MOCAORYR
MOCACLON
SEX
MOCACUBE
−1.5
−1.0
−0.5
0.0
0.5
1.0
1.5
SHAP value (impact on model output)
High
Low
Feature value

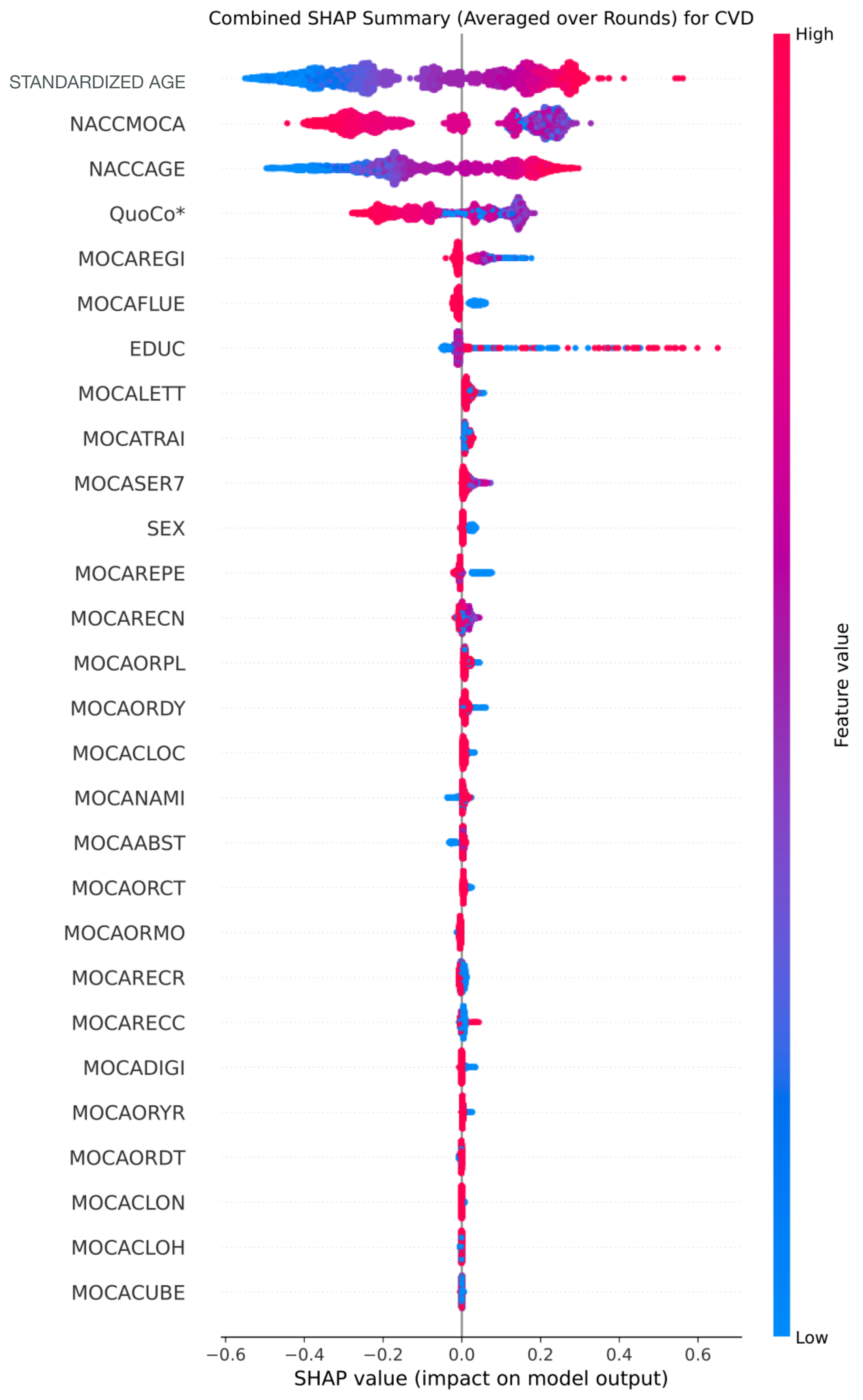
Combined SHAP Summary (Averaged over Rounds) for CVD
STANDARDIZED AGE
NACCMOCA
NACCAGE
QuoCo*
MOCAREGI
MOCAFLUE
EDUC
MOCALETT
MOCATRAI
MOCASER7
SEX
MOCAREPE
MOCARECN
MOCAORPL
MOCAORDY
MOCACLOC
MOCANAMI
MOCAABST
MOCAORCT
MOCAORMO
MOCARECR
MOCARECC
MOCADIGI
MOCAORYR
MOCAORDT
MOCACLON
MOCACLOH
MOCACUBE
−0.6
−0.4
−0.2
0.0
0.2
0.4
0.6
SHAP value (impact on model output)
High
Low
Feature value

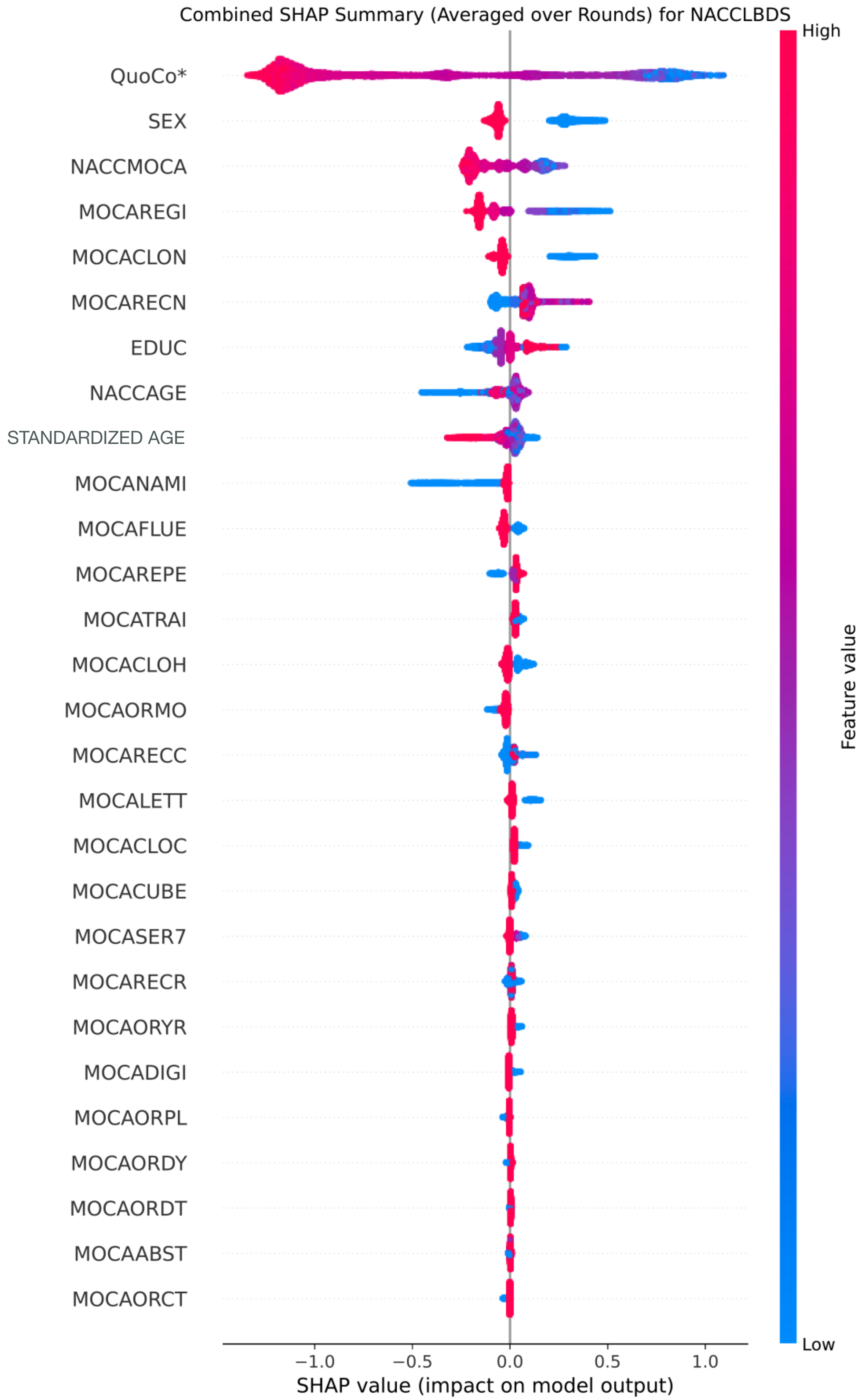

Combined SHAP Summary (Averaged over Rounds) for NACCLBDS
QuoCo*
SEX
NACCMOCA
MOCAREGI
MOCACLON
MOCARECN
EDUC
NACCAGE
STANDARDIZED AGE
MOCANAMI
MOCAFLUE
MOCAREPE
MOCATRAI
MOCACLOH
MOCAORMO
MOCARECC
MOCALETT
MOCACLOC
MOCACUBE
MOCASER7
MOCARECR
MOCAORYR
MOCADIGI
MOCAORPL
MOCAORDY
MOCAORDT
MOCAABST
MOCAORCT
−1.0
−0.5
0.0
0.5
1.0
SHAP value (impact on model output)
High
Low
Feature value

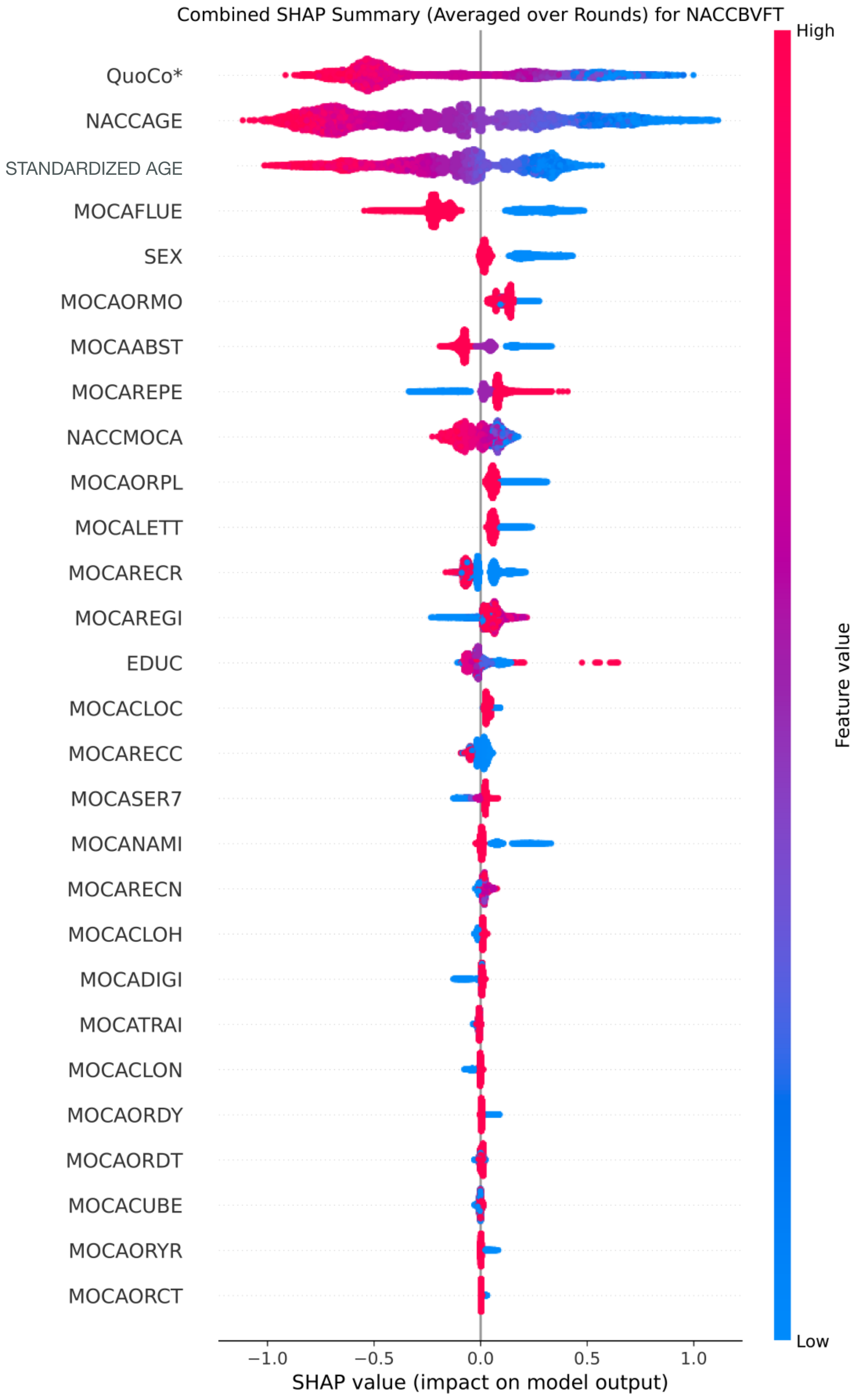
Combined SHAP Summary (Averaged over Rounds) for NACCBVFT
QuoCo*
NACCAGE
STANDARDIZED AGE
MOCAFLUE
SEX
MOCAORMO
MOCAABST
MOCAREPE
NACCMOCA
MOCAORPL
MOCALETT
MOCARECR
MOCAREGI
EDUC
MOCACLOC
MOCARECC
MOCASER7
MOCANAMI
MOCARECN
MOCACLOH
MOCADIGI
MOCATRAI
MOCACLON
MOCAORDY
MOCAORDT
MOCACUBE
MOCAORYR
MOCAORCT
−1.0
−0.5
0.0
0.5
1.0
SHAP value (impact on model output)
High
Low
Feature value

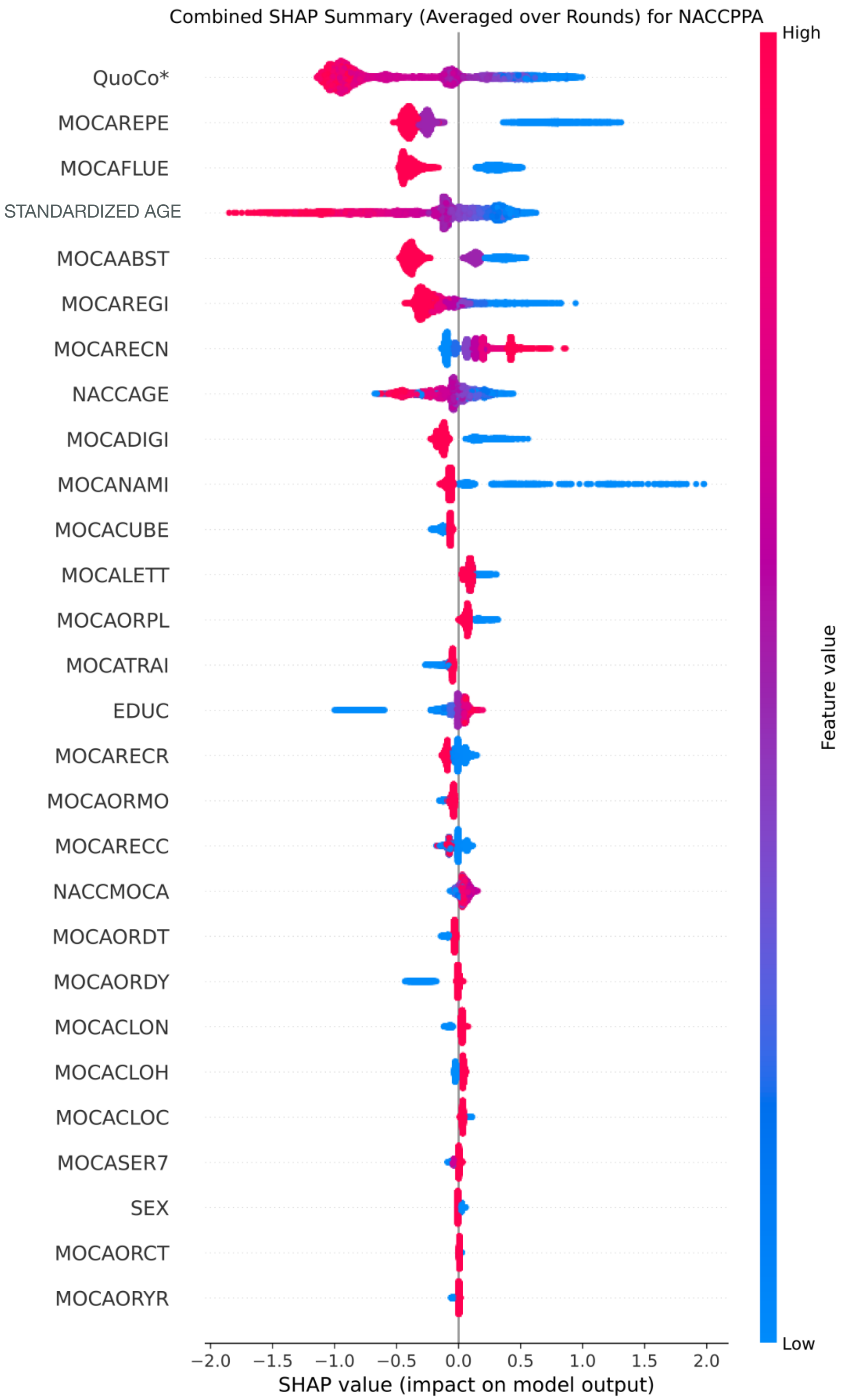

Combined SHAP Summary (Averaged over Rounds) for NACCPPA
QuoCo*
MOCAREPE
MOCAFLUE
STANDARDIZED AGE
MOCAABST
MOCAREGI
MOCARECN
NACCAGE
MOCADIGI
MOCANAMI
MOCACUBE
MOCALETT
MOCAORPL
MOCATRAI
EDUC
MOCARECR
MOCAORMO
MOCARECC
NACCMOCA
MOCAORDT
MOCAORDY
MOCACLON
MOCACLOH
MOCACLOC
MOCASER7
SEX
MOCAORCT
MOCAORYR
−2.0 −1.5 −1.0 −0.5 0.0 0.5 1.0 1.5 2.0
SHAP value (impact on model output)
High
Feature value
Low

Demographic & Global Metrics

- **QuoCo:** Cognitive Quotient (Derived metric indicating cognitive performance relative to age/education)
- **STANDARDIZED AGE:** Age adjusted for education level
- **NACCMOCA:** Total MoCA Score (0–30)
- **NACCAGE:** Chronological Age
- **EDUC:** Years of Education
- **SEX:** Sex

Memory (Recall & Registration)

- **MOCAREGI:** Immediate Registration (Learning trials)
- **MOCARECN:** Delayed Recall – No Cue (Free recall)
- **MOCARECC:** Delayed Recall – Category Cue
- **MOCARECR:** Delayed Recall – Recognition (Multiple choice)

Orientation

- **MOCAORDT:** Orientation – Date
- **MOCAORMO:** Orientation – Month
- **MOCAORYR:** Orientation – Year
- **MOCAORDY:** Orientation – Day
- **MOCAORPL:** Orientation – Place
- **MOCAORCT:** Orientation – City
-

Visuospatial & Executive Function

- **MOCATRAI:** Trail Making (Alternating Trail Making)
- **MOCACUBE:** Copy Cube
- **MOCACLOC:** Clock Drawing – Contour
- **MOCACLON:** Clock Drawing – Numbers
- **MOCACLOH:** Clock Drawing – Hands

Attention

- **MOCADIGI:** Digit Span (Forward and Backward)
- **MOCALETT:** Letter "A" Tapping
- **MOCASER7:** Serial 7 Subtraction

Language

- **MOCANAMI:** Naming
- **MOCAREPE:** Sentence Repetition
- **MOCAFLUE:** Verbal Fluency (Letter F)

Abstraction

- **MOCAABST:** Abstraction (Similarity between words)
