# Supplementary material for: Enhanced diagnostic interpretation of the MoCA using machine learning
Source: Front Neurosci. 2026 Feb 20;20:1679649. doi: 10.3389/fnins.2026.1679649 (PMC12963294; doi:10.3389/fnins.2026.1679649)
Supplement: Supplementary file 2 [file Data_Sheet_2.docx]

## Supplementary Table 4.

## SHAP Importance Ranking

Variables are ordered by their mean SHAP rank across all diagnostic categories. Ranks range from 1 (most influential) to 28 (least influential). Cell shading reflects rank intensity (white = highest importance, darker = lower importance).

| Variable | Description | Global | Alzheimer | Vascular | Lewy Body | bvFTD | PPA |
| --- | --- | --- | --- | --- | --- | --- | --- |
| QuoCo* | Cognitive Quotient | 1 | 4 | 4 | 1 | 1 | 1 |
| NACCMOCA | Total MoCA score | 2 | 2 | 2 | 3 | 9 | 19 |
| NACCAGE | Chronological age | 13 | 8 | 3 | 8 | 2 | 8 |
| Standardized age | Age adjusted for education | 17 | 9 | 1 | 9 | 3 | 4 |
| MOCAREGI | Immediate registration | 9 | 10 | 5 | 4 | 13 | 6 |
| MOCARECN | Uncued delayed recall | 3 | 1 | 13 | 6 | 19 | 7 |
| MOCAFLUE | Verbal fluency | 20 | 11 | 6 | 11 | 4 | 3 |
| MOCAORMO | Orientation month | 4 | 5 | 20 | 15 | 6 | 17 |
| EDUC | Education years | 15 | 14 | 7 | 7 | 14 | 15 |
| MOCAORPL | Orientation place | 6 | 6 | 14 | 24 | 10 | 13 |
| MOCALETT | Letter tapping | 14 | 13 | 8 | 17 | 11 | 12 |
| MOCAREPE | Repetition | 22 | 20 | 12 | 12 | 8 | 2 |
| SEX | Sex | 12 | 27 | 11 | 2 | 5 | 26 |
| MOCATRAI | Trail making | 11 | 22 | 9 | 13 | 22 | 14 |
| MOCARECC | Cued recall | 10 | 12 | 22 | 16 | 16 | 18 |
| MOCAORDY | Orientation day | 5 | 7 | 15 | 25 | 24 | 21 |
| MOCAABST | Abstraction | 25 | 15 | 18 | 27 | 7 | 5 |
| MOCARECR | Recognition recall | 18 | 17 | 21 | 21 | 12 | 16 |
| MOCANAMI | Naming | 26 | 24 | 17 | 10 | 18 | 10 |
| MOCAORDT | Orientation date | 7 | 3 | 25 | 26 | 25 | 20 |
| MOCASER7 | Serial subtraction | 19 | 19 | 10 | 20 | 17 | 25 |
| MOCACLOC | Clock drawing contour | 23 | 21 | 16 | 18 | 15 | 24 |
| MOCACLON | Clock drawing numbers | 21 | 26 | 26 | 5 | 23 | 22 |
| MOCADIGI | Digit span | 24 | 23 | 23 | 23 | 21 | 9 |
| MOCACLOH | Clock drawing hands | 27 | 16 | 27 | 14 | 20 | 23 |
| MOCAORYR | Orientation year | 8 | 25 | 24 | 22 | 27 | 28 |
| MOCAORCT | Orientation city | 16 | 18 | 19 | 28 | 28 | 27 |
| MOCACUBE | Cube copying | 28 | 28 | 28 | 19 | 26 | 11 |
