# Supplementary material for: Enhanced diagnostic interpretation of the MoCA using machine learning
Source: Front Neurosci. 2026 Feb 20;20:1679649. doi: 10.3389/fnins.2026.1679649 (PMC12963294; doi:10.3389/fnins.2026.1679649)

## Slide 1
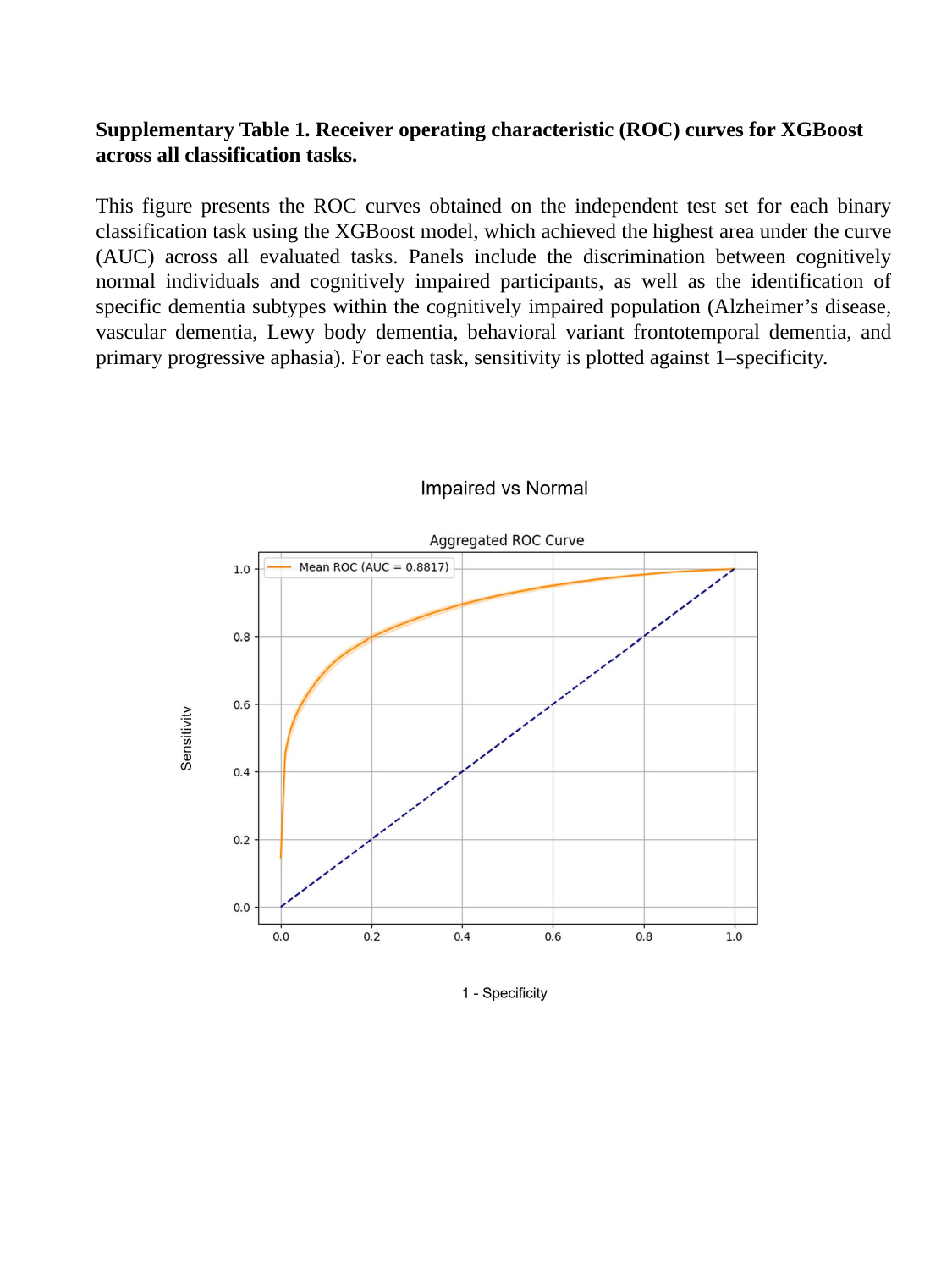

Supplementary Table 1. Receiver operating characteristic (ROC) curves for XGBoost across all classification tasks.
This figure presents the ROC curves obtained on the independent test set for each binary classification task using the XGBoost model, which achieved the highest area under the curve (AUC) across all evaluated tasks. Panels include the discrimination between cognitively normal individuals and cognitively impaired participants, as well as the identification of specific dementia subtypes within the cognitively impaired population (Alzheimer’s disease, vascular dementia, Lewy body dementia, behavioral variant frontotemporal dementia, and primary progressive aphasia). For each task, sensitivity is plotted against 1–specificity.

## Slide 2
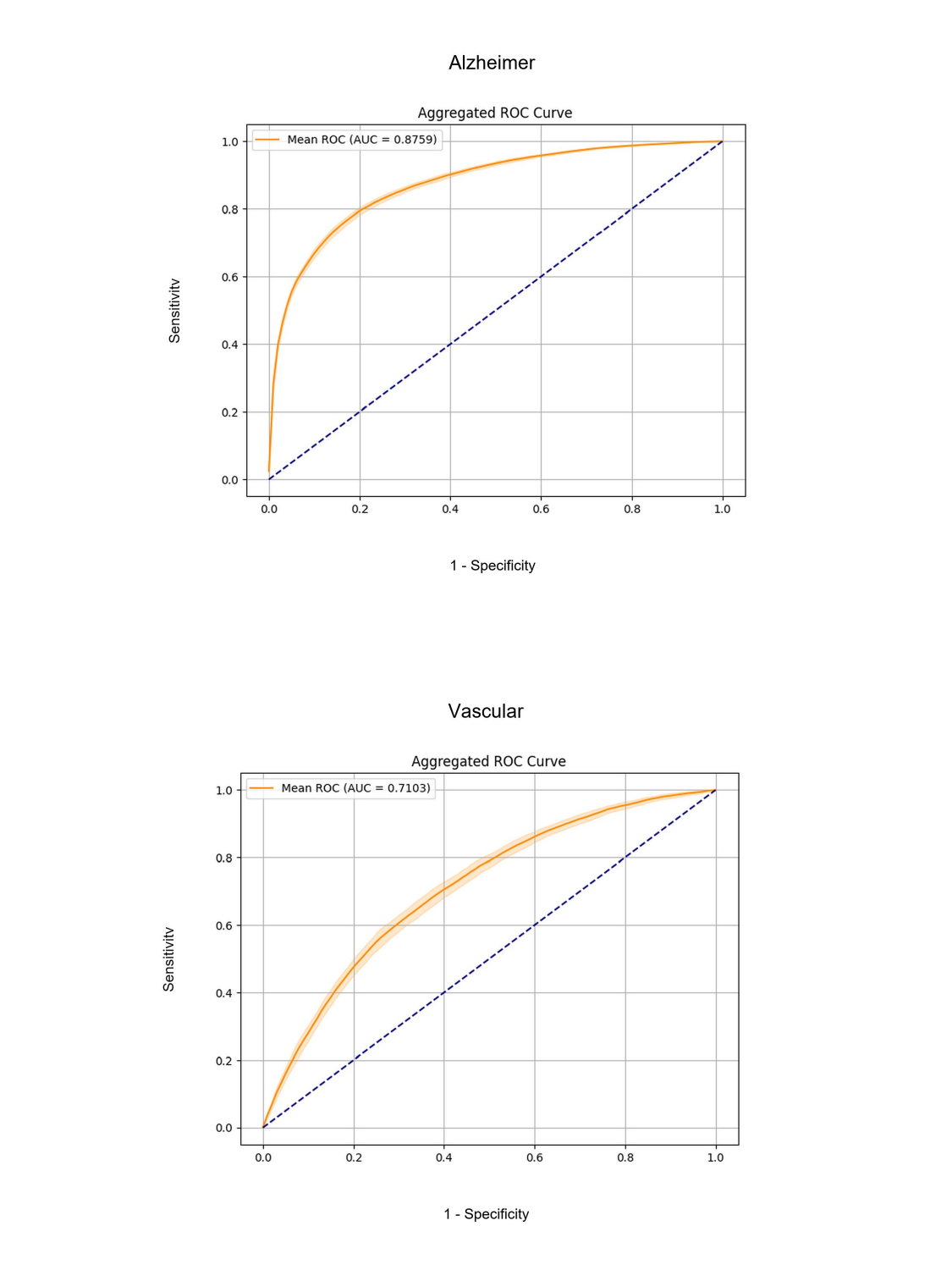

## Slide 3
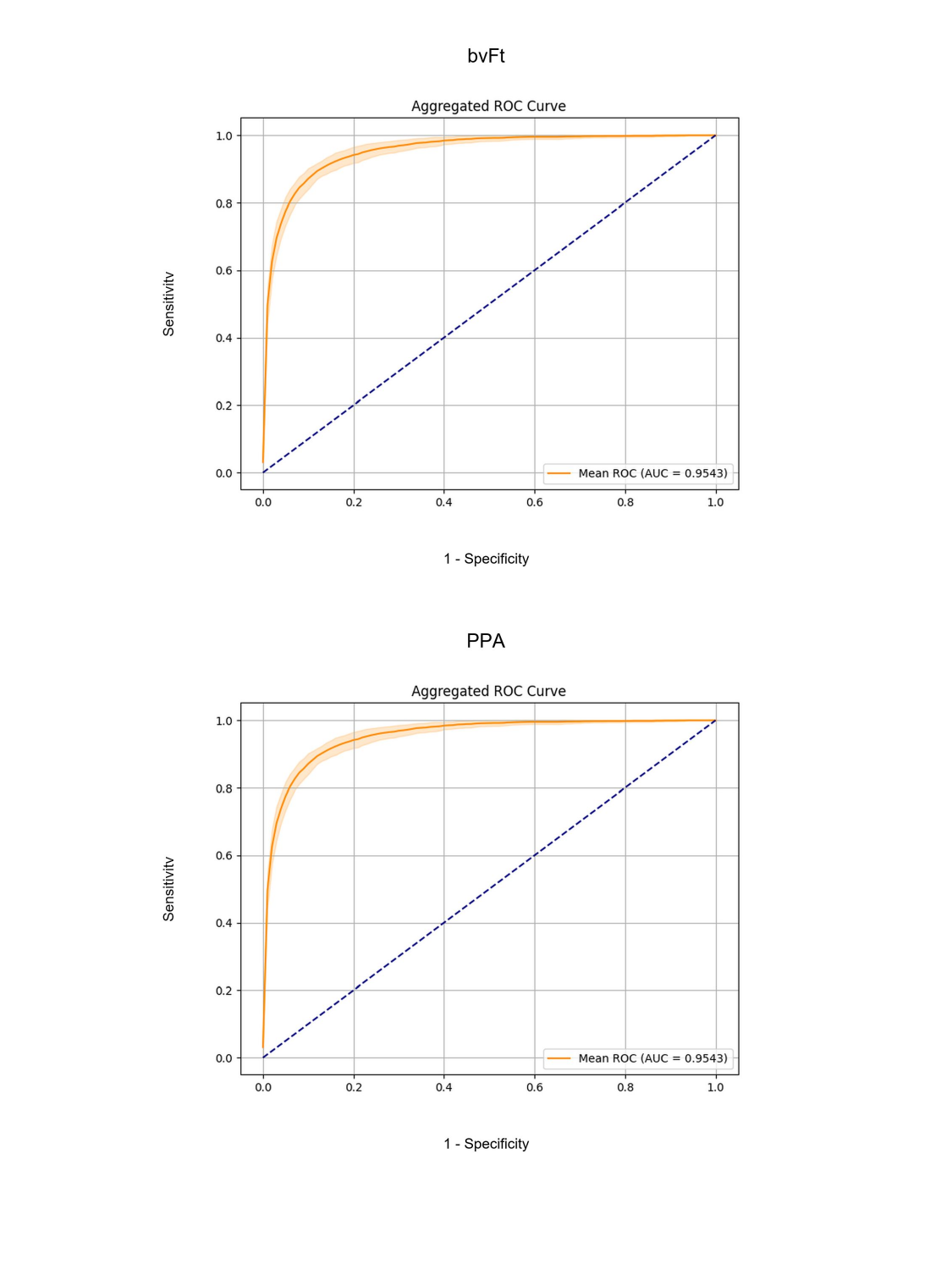

## Slide 4
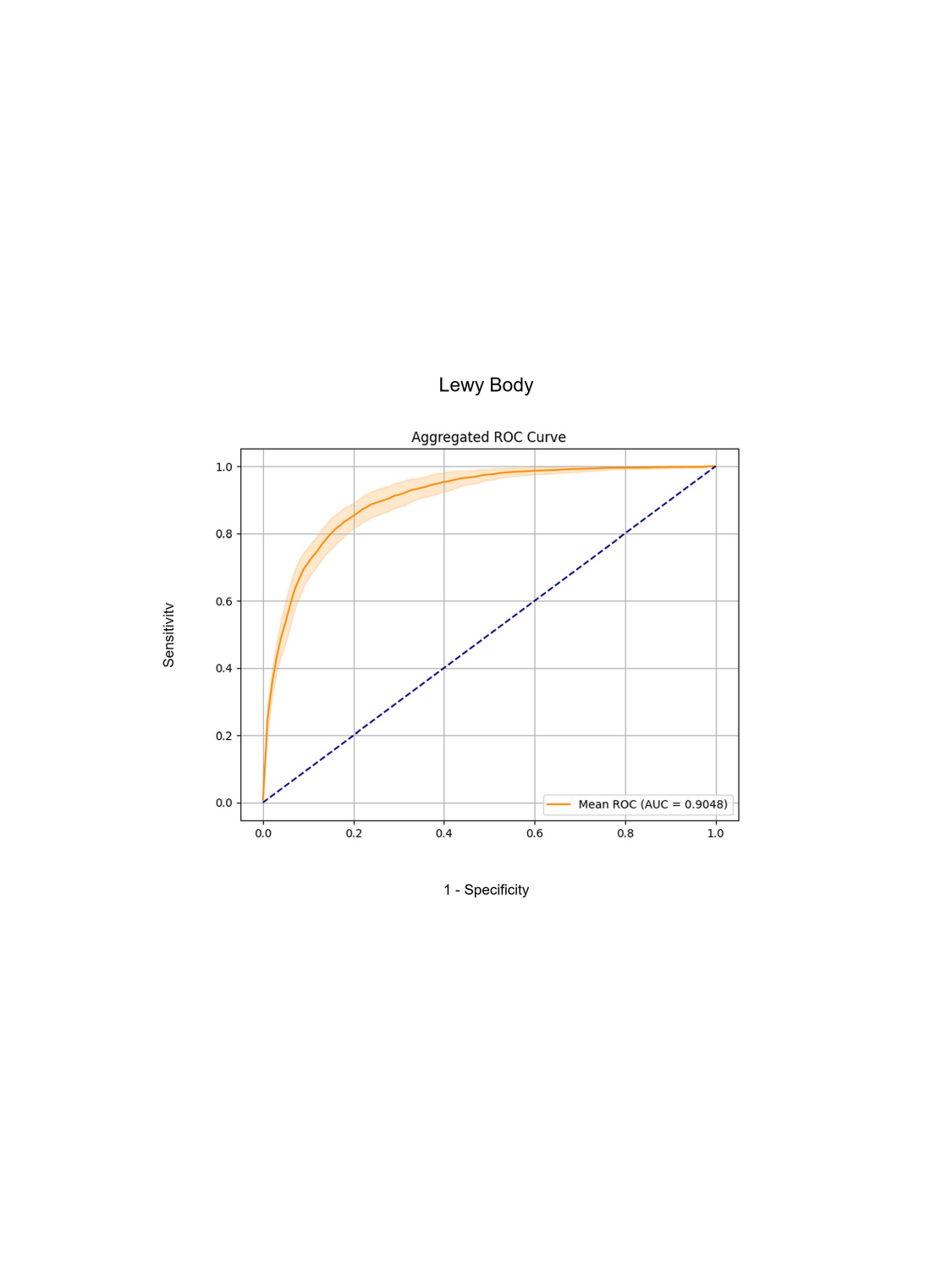

Supplement: Supplementary file 3 [file Presentation_1.pptx]
