# Supplementary material for: Enhanced diagnostic interpretation of the MoCA using machine learning
Source: Front Neurosci. 2026 Feb 20;20:1679649. doi: 10.3389/fnins.2026.1679649 (PMC12963294; doi:10.3389/fnins.2026.1679649)

## Slide 1
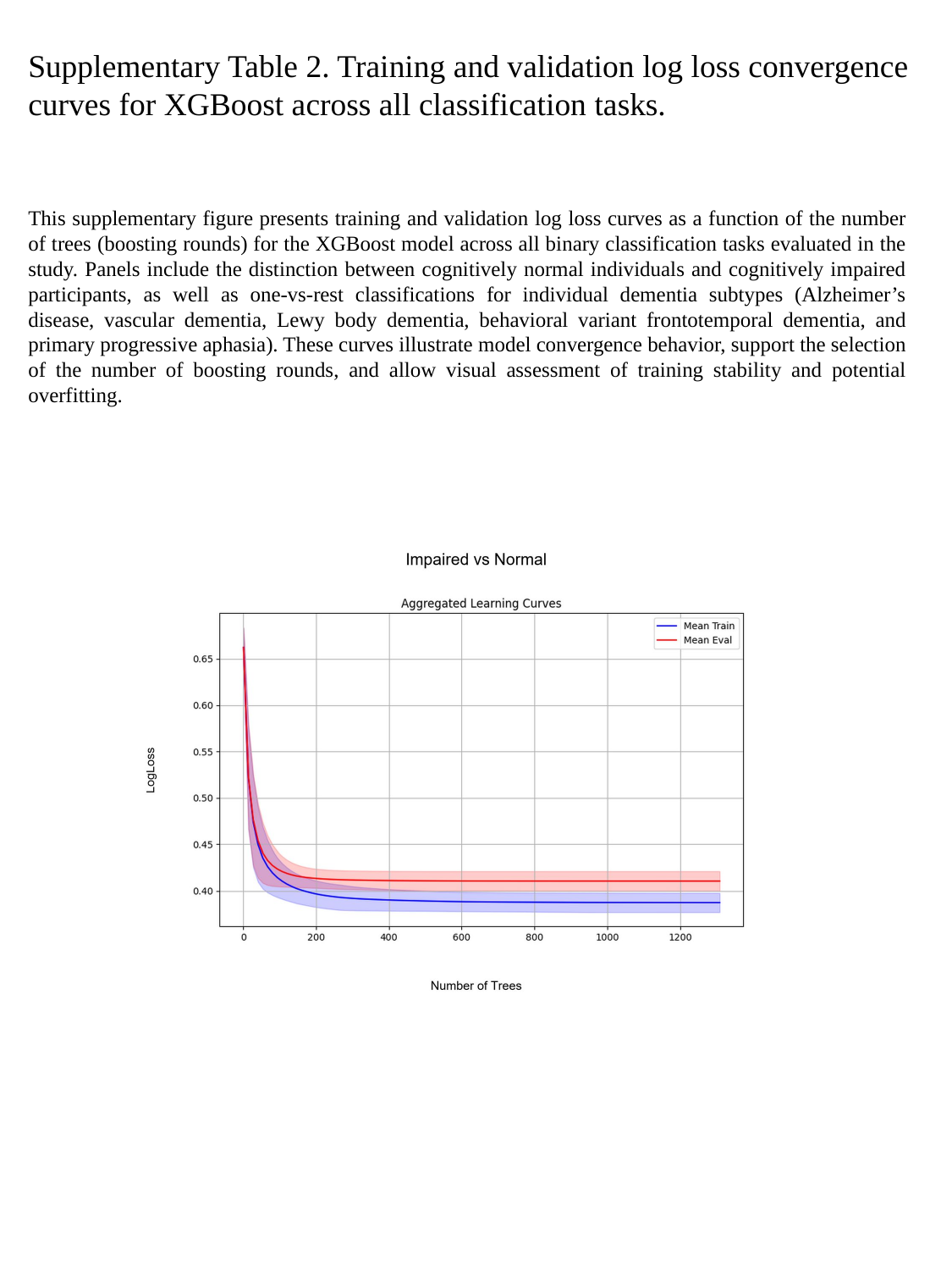

Supplementary Table 2. Training and validation log loss convergence curves for XGBoost across all classification tasks.
This supplementary figure presents training and validation log loss curves as a function of the number of trees (boosting rounds) for the XGBoost model across all binary classification tasks evaluated in the study. Panels include the distinction between cognitively normal individuals and cognitively impaired participants, as well as one-vs-rest classifications for individual dementia subtypes (Alzheimer’s disease, vascular dementia, Lewy body dementia, behavioral variant frontotemporal dementia, and primary progressive aphasia). These curves illustrate model convergence behavior, support the selection of the number of boosting rounds, and allow visual assessment of training stability and potential overfitting.

## Slide 2
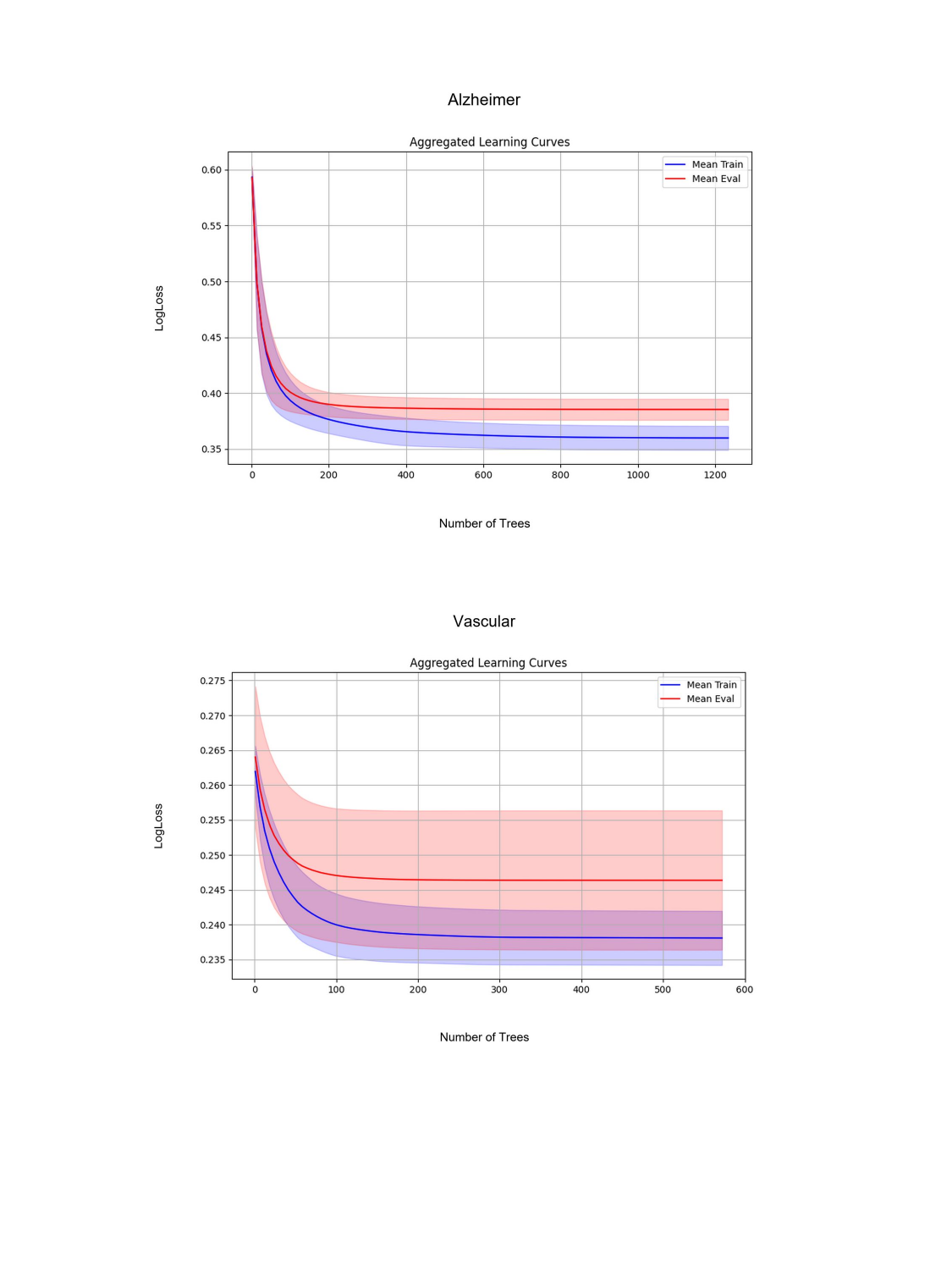

## Slide 3
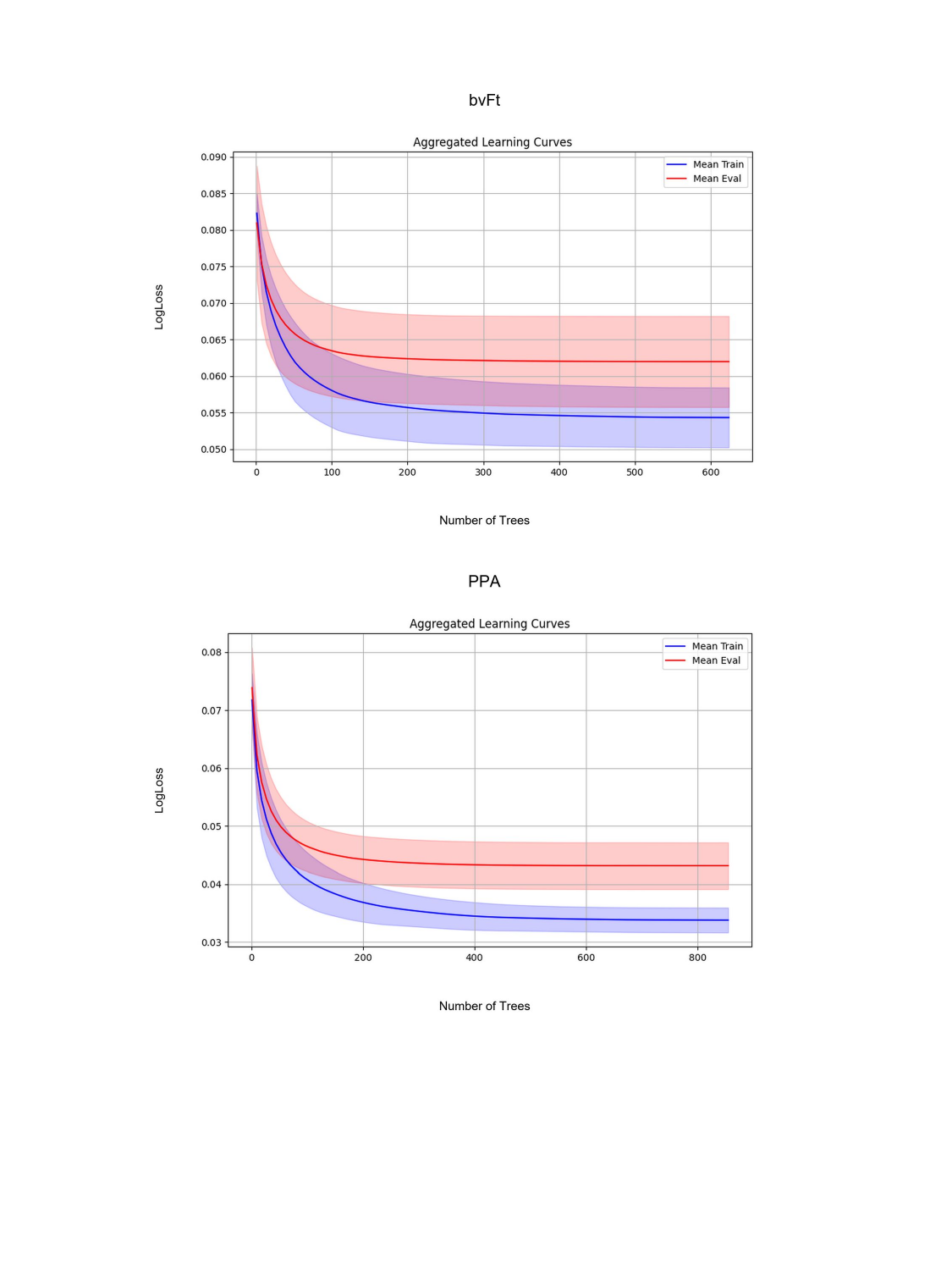

## Slide 4
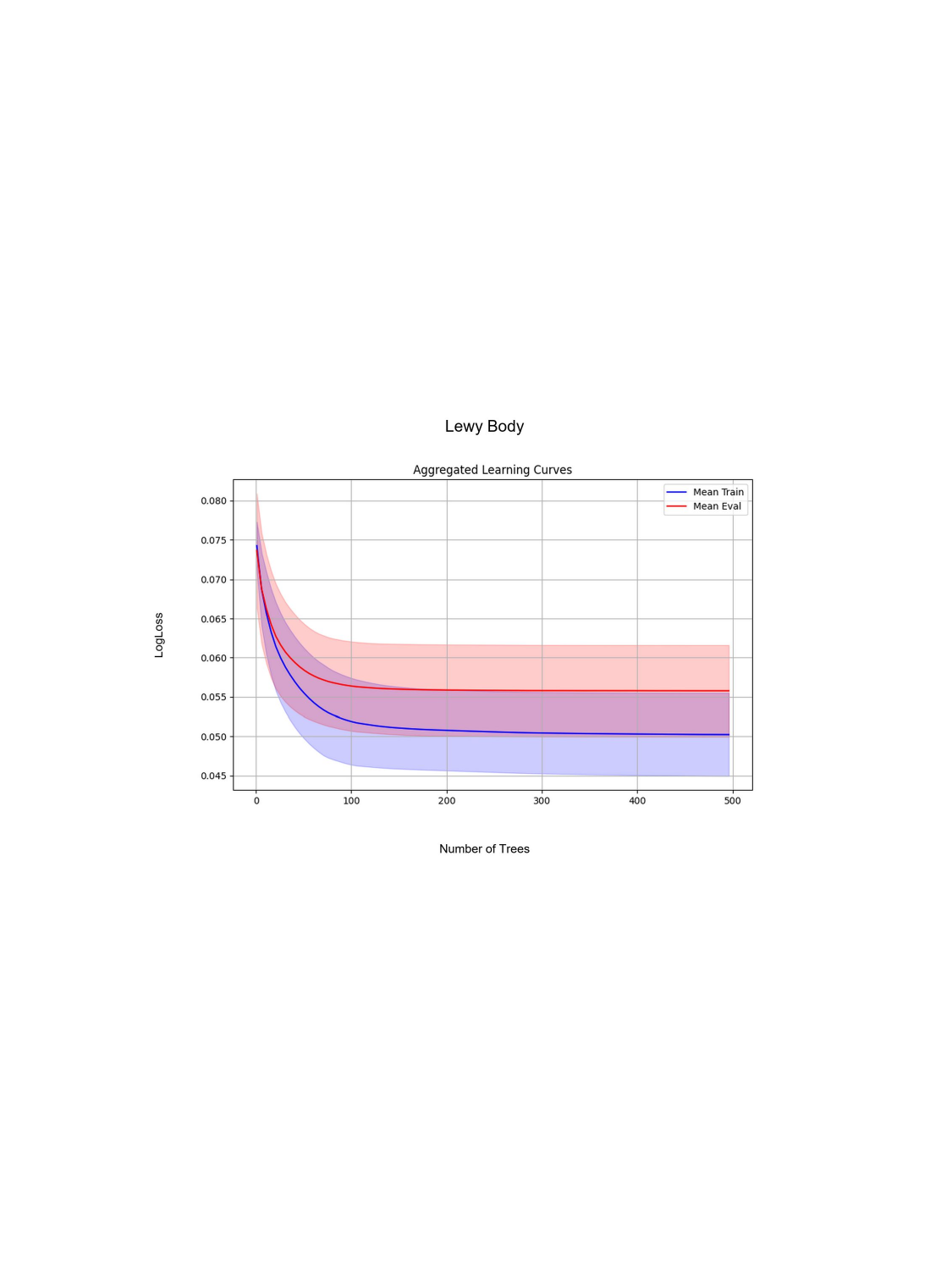

Supplement: Supplementary file 4 [file Presentation_2.pptx]
